# Characterization of SARS-CoV-2 Spike mutations important for infection of mice and escape from human immune sera

**DOI:** 10.1038/s41467-022-30763-0

**Published:** 2022-07-07

**Authors:** Raveen Rathnasinghe, Sonia Jangra, Chengjin Ye, Anastasija Cupic, Gagandeep Singh, Carles Martínez-Romero, Lubbertus C. F. Mulder, Thomas Kehrer, Soner Yildiz, Angela Choi, Stephen T. Yeung, Ignacio Mena, Virginia Gillespie, Jana De Vrieze, Sadaf Aslam, Daniel Stadlbauer, David A. Meekins, Chester D. McDowell, Velmurugan Balaraman, Michael J. Corley, Juergen A. Richt, Bruno G. De Geest, Lisa Miorin, PVI study group, Giulio Kleiner, Miti Saksena, Komal Srivastava, Charles R. Gleason, Maria C. Bermúdez-González, Katherine F. Beach, Kayla T. Russo, Levy A. Sominsky, Emily D. Ferreri, Rachel L. Chernet, Lily Q. Eaker, Ashley-Beathrese T. Salimbangon, Denise Jurczyszak, Hala Alshammary, Wanni A. Mendez, Angela A. Amoako, Shelcie Fabre, Mahmoud H. Awawda, Amber S. Shin, Florian Krammer, Luis Martinez-Sobrido, Viviana Simon, Adolfo García-Sastre, Michael Schotsaert

**Affiliations:** 1 Department of Microbiology, Icahn School of Medicine at Mount Sinai New York, New York, NY USA; 2 Graduate School of Biomedical Sciences, Icahn School of Medicine at Mount Sinai, New York, NY USA; 3 Global Health and Emerging Pathogens Institute, Icahn School of Medicine at Mount Sinai New York, New York, NY USA; 4 Texas Biomedical Research Institute, San Antonio, TX USA; 5 Division of Infectious Diseases, Department of Medicine, Weill Cornell Medicine, New York, New York, NY USA; 6 Center for Comparative Medicine and Surgery, Icahn School of Medicine at Mount Sinai New York, New York, NY USA; 7 Department of Pharmaceutics, Ghent University, Ghent, Belgium; 8 Department of Diagnostic Medicine/Pathobiology, College of Veterinary Medicine, Kansas State University, Manhattan, KS USA; 9 Department of Medicine, Division of Infectious Diseases, Icahn School of Medicine at Mount Sinai New York, New York, NY USA; 10 The Tisch Cancer Institute, Icahn School of Medicine at Mount Sinai New York, New York, NY USA; 11 Department of Microbiology, Icahn School of Medicine at Mount Sinai New York, New York, NY USA; 12 Global Health and Emerging Pathogens Institute, Icahn School of Medicine at Mount Sinai New York, New York, NY USA; 13 Present Address: Seqirus, Cambridge, MT USA; 14 Present Address: Moderna Therapeutics, Cambridge, MT USA

**Keywords:** SARS-CoV-2, Infectious diseases

## Abstract

Due to differences in human and murine angiotensin converting enzyme 2 (ACE-2) receptor, initially available SARS-CoV-2 isolates could not infect mice. Here we show that serial passaging of USA-WA1/2020 strain in mouse lungs results in “mouse-adapted” SARS-CoV-2 (MA-SARS-CoV-2) with mutations in S, M, and N genes, and a twelve-nucleotide insertion in the S gene. MA-SARS-CoV-2 infection causes mild disease, with more pronounced morbidity depending on genetic background and in aged and obese mice. Two mutations in the S gene associated with mouse adaptation (N501Y, H655Y) are present in SARS-CoV-2 variants of concern (VoCs). N501Y in the receptor binding domain of viruses of the B.1.1.7, B.1.351, P.1 and B.1.1.529 lineages (Alpha, Beta, Gamma and Omicron variants) is associated with high transmissibility and allows VoCs to infect wild type mice. We further show that S protein mutations of MA-SARS-CoV-2 do not affect neutralization efficiency by human convalescent and post vaccination sera.

## Abstract (web summary)

Here, Rathnasinghe *et al*. characterize the pathogenesis of a mouse-adapted SARS-CoV-2 in infected obese and aged mice, and identify mutations that are present in SARS-CoV-2 variants of concern, associated with higher transmissibility.

## Introduction

Severe acute respiratory syndrome coronavirus-2 (SARS-CoV-2) is the cause of the present coronavirus disease 2019 (COVID-19) pandemic, which has claimed hundreds of thousands of lives with the death toll still rising. The virus belongs to the family *Coronaviridae* and genera Betacoronaviruses which consists of a single-stranded, positive sense ~30 kB RNA as genome. The genome encodes for four structural proteins—nucleoprotein (N), spike (S), envelope (E), and membrane (M) proteins. The S protein of the virus plays an integral part in viral fusion and entry into host cells. The homotrimeric S protein consists of S1 and S2 subunits. The receptor-binding domain (RBD) of the S1 subunit binds to angiotensin-converting enzyme 2 (ACE-2) present on the host cellular surfaces. The interaction is followed by S2-driven-transcleavage of the S protein by cellular metalloproteases such as TMPRSS2, thereby facilitating an efficient fusion and release of viral contents into the host cell^1^. Since the RBD is necessary for direct interaction with the host receptor, mutations in RBD can affect SARS-CoV-2 infection efficiency depending on the host. As the S RBD is also the target of virus-neutralizing antibodies, mutations in RBD can impact the neutralizing titers of polyclonal and monoclonal antibodies.

SARS-CoV-2 primarily spreads through respiratory droplets and causes a diverse array of symptoms from completely asymptomatic infections to fever, cough, anosmia, pneumonia, acute respiratory distress syndrome, microvascular coagulation, multi-organ dysfunction, and other severe manifestations—including death—in humans. Several predisposition factors have been found to be associated with increased susceptibility to severe COVID-19. Hypertension, cardiovascular diseases, gender, advanced age, and obesity have already been defined as major risk factors among humans attributing to increased mortality by enhancing secondary conditions such as hypoxemia and pneumonia^2–7^. In order to study SARS-CoV-2-associated comorbidities, there is a need for small animal models to study the effect of host conditions on the outcome of SARS-CoV-2 infection. The RBD of the S protein from the SARS-CoV-2 strain that started the pandemic does not efficiently bind mouse ACE-2 (mACE-2, a murine ortholog of human (h)-ACE-2)^8^ and as a consequence this SARS-CoV-2 strain does not efficiently infect laboratory mouse strains. Several approaches have been developed to allow the use of mouse models for SARS-CoV-2 research^9–11^, often guided by the experience obtained previously for mouse models to study SARS-CoV. Many of these models rely on transgenic mice that express hACE-2 in epithelial cells or sensitize mice to SARS-CoV-2 infection by adenovirus-mediated transduction of the hACE-2 gene (Ad-hACE-2) in the respiratory tract^9,12^. These models have been crucial for studying host-pathogen interactions, prophylactic, and therapeutic interventions in the context of SARS-CoV and SARS-CoV-2 infection. However, a major drawback of these models is that expression of hACE-2 is often driven by a promoter that is not the original ACE-2 promoter, and therefore promoter control and expression patterns can differ, and, in the case of Ad-hACE-2, depend on transduction efficiency. These problems would be circumvented by a mouse-adapted SARS-CoV-2 (MA-SARS-CoV-2) that uses the endogenously expressed mACE-2. Moreover, a MA-SARS-CoV-2 can be used with already established mouse models of comorbidities associated with more severe COVID-19 and allows the efficient exploitation of the genetic toolboxes available for mice. Several efforts were undertaken to generate MA-SARS-CoV-2^13–16^.

In this study, we have developed and characterized a MA-SARS-CoV-2 strain after serially passaging a clinical virus isolate (USA-WA1/2020) first in immune-compromised followed by immune-competent mice. We mapped mutations associated with mouse adaptation in the SARS-CoV-2 genome and observed that one of them is the N501Y mutation in the viral S RBD that is also reported for some of the SARS-CoV-2 variants of concern (VoC) that started dominating during the COVID-19 pandemic (B.1.1.7, B.1.351, P.1, and B.1.1.529, also called Alpha, Beta, Gamma, and Omciron variants, respectively, according to WHO labeling) with enhanced human transmission potential^17^. We show that N501Y VoCs can directly infect laboratory mice as well. We used the mouse-adapted SARS-CoV-2 strain with N501Y mutation to show that SARS-CoV-2 infection comes with enhanced morbidity in mouse models for advanced age, obesity, and obesity-associated type 2 diabetes mellitus. When focusing on the effect of individual mutations observed in VOC spike proteins on escape from virus neutralization by human sera, we show that human sera from convalescent and vaccinated individuals can neutralize both the reference USA-WA1/2020 strain and the mouse-adapted strain that contains the N501Y S mutation with similar efficiency suggesting that other mutations in the VoC S proteins are responsible for immune escape. Moreover, using human convalescent and post-vaccination sera, we observe that vaccination resulted in high neutralizing antibody titers for all viruses tested in this study, where the titers were comparatively higher for individuals that experienced COVID-19 before vaccination and immune escape by VoCs and recombinant viruses were more reduced in post-vaccination sera compared to convalescent sera.

## Results

### Serial passaging of SARS-CoV-2 in mice results in mouse-adapted SARS-CoV-2

**Fig. 1:**
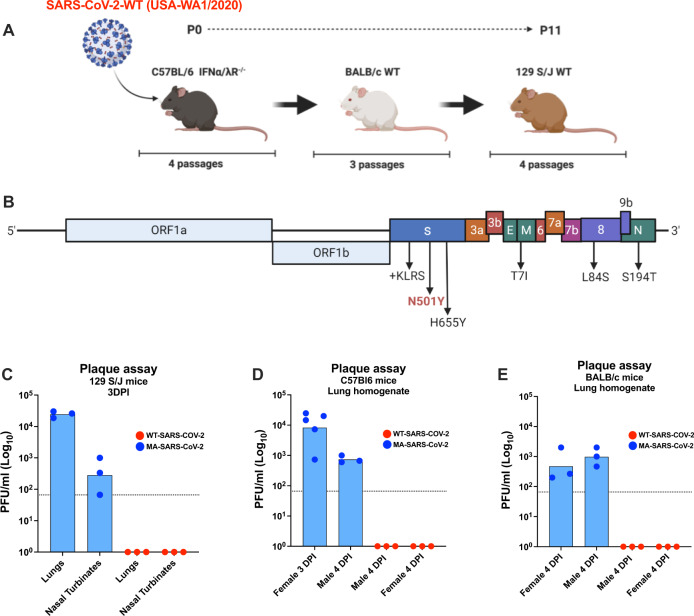
Mouse-adaptation strategy and lung virus titers upon challenge with WT- and MA-SARS-CoV-2. **A** The SARS-CoV-2 USA-WA1/2020 Seattle strain was obtained from BEI Resources and serially passaged 11 times in mice of different genetic backgrounds. **B** Sequence and location of mutations identified in MA-SARS-CoV-2 when compared to WT-SARS-CoV-2. **C** Infection with 2.5 × 10^4^ PFU of the MA-SARS-CoV-2 resulted in detectable virus titers in lungs (right lung lobes were used for titration) and nasal turbinates at 3 DPI in female 129S1 mice whereas the WT-SARS-CoV-2 did not. **D** Infection with 2.5 × 10^4^ PFU of MA-SARS-CoV-2 but not WT-SARS-CoV-2, resulted in detectable lung virus titers harvested at different time points post-infection in C57Bl6 as well as **E** BALB/c mice irrespective of sex. Bars represent geometric means. Dotted line indicates limit of detection (LOD) −66.67 PFUml^−1^. Each data point corresponds to one mouse and the number of data points represents the number of mice in the corresponding groups. Source data are provided as a Source Data file.

The USA-WA1/2020-SARS-CoV-2 (termed WT-SARS-CoV-2) virus isolate was passaged eleven times in the lungs of various strains of mice as outlined in Fig. 1A. The virus was first allowed to adapt to mACE-2 receptor in immune-compromised mice with weakened innate immune responses. To this end, the virus was consecutively passaged four times in IFNα/λ receptor knock-out mice in C57BL6 genetic background, using 50 µl of lung homogenate from each infected mouse collected 3 days post-infection (DPI). The virus was then further passaged three times in BALB/c mice and four times in 129S1 mice. The 129S1 mice were chosen for mouse adaptation as they have been shown to be more susceptible to other viruses as compared to other laboratory mouse strains, probably due to mouse strain-specific differences in immune cell distribution^18–20^. After 11 passages, the virus was plaque-purified, and clonal virus stocks of the MA-SARS-CoV-2 were prepared in mACE-2 expressing Vero-E6 for further infection experiments. For comparative purposes, clonal virus stocks of the WT-SARS-CoV-2 were also generated using Vero-E6 cells. No differences in growth kinetics were observed for MA-SARS-CoV-2 on Vero-E6 cells (See Supplementary Fig. 2), confirming that MA-SARS-CoV-2 can also use the primate ACE2 similar to the original USA-WA1/2020. The consensus genomic sequence of the MA-SARS-CoV-2 was generated by Sanger and deep sequencing methods and the sequence changes compared to the original SARS-CoV-2/human/Wuhan/X1/2019 genome sequence (NC_045512.3) are summarized in Fig. 1B. The MA-SARS-CoV-2 contained two amino-acid mutations and a four amino-acid insertion (KLRS) after residue 215 in the S, and one amino-acid mutation in the M, and N gene products of the virus (Fig. 1B). One of the amino-acid changes in the RBD of S protein in the virus, N501Y, has previously been reported to be associated with mouse-adaptation of SARS-CoV-2^15^ and is predicted to increase binding to mACE-2^21^. Interestingly, the same N501Y mutation has been reported in newly emerging SARS-CoV-2 VoCs (B.1.1.7, B.1.351, P.1, B.1.1.529) with the potentially enhanced human transmission. A second mutation that is present in VoCs P.1 and B.1.1.529 was observed close to the furin cleavage site (H655Y). In unrelated experiments performed at Kansas State University, the KLRS insertion was also observed in viruses recovered from cats infected with USA-WA1/2020 (Supplementary Fig. 3). The L84S mutation observed in ORF8 was already present in the USA-WA1/2020 virus isolate from which the MA-SARS-CoV-2 was started. Additionally, mutations in N (S194T) and M (T7I) were also observed.

### MA-SARS-CoV-2 efficiently replicates in the lungs of different wild-type strains of mice and results in transient morbidity in 129S1 mice

**Fig. 2:**
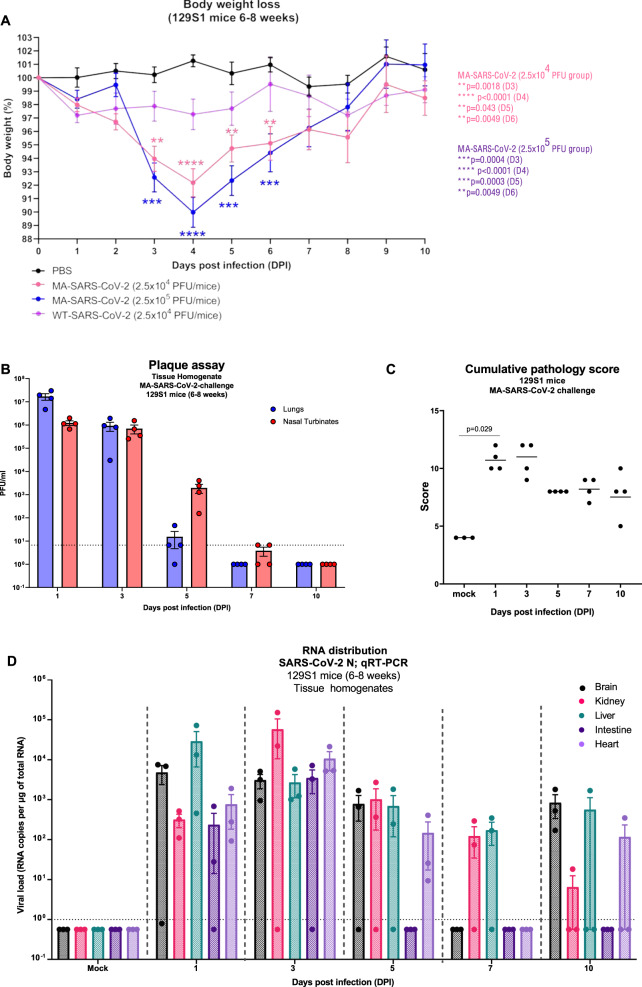
Morbidity, viral replication, pathology, and tissue distribution of viral RNA after MA-SARS-CoV-2 infection of 129S1 mice. **A** Evolution of body weight relative to initial body weight after infection with 2.5 × 10^4^ PFU WT-SARS-CoV-2 (*n* = 5), PBS/mock (*n* = 3), and two doses of MA-SARS-CoV-2 (*n* = 20 for 2.5 × 10^4^ PFU, *n* = 5 for 2.5 × 10^5^ PFU). Differences were compared to PBS group. Each dot represents individual animal and the error bar represents mean ± SEM. Two-sided unpaired *t*-test was performed to determine the statistical difference in reference to the mock group at different DPI. **B** Viral load in whole lungs and nasal turbinates during the course of infection with 2.5 × 10^4^ PFU of MA-SARS-CoV-2 as measured by plaque assay (*n* = 4). PFU = plaque-forming units. **C** Cumulative pathology scored during the course of infection with 2.5 × 10^4^ PFU of MA-SARS-CoV-2 (*n* = 3 for mock; *n* = 4 for other groups). Each dot represents individual animal, and the bar represents geometric mean in respective group. Two-tailed Mann–Whitney U test was performed to determine the statistical difference between mock and 1DPI. **D** Genomic RNA copies in different tissues at different time points post-infection with 2.5 × 10^4^ PFU of MA-SARS-CoV-2 quantified by qRT-PCR using primers specific for the N gene (*n* = 3). Two-sided unpaired *t*-test was performed to calculate the statistical significance between different groups. Bars represent geometric means; error bars represent standard deviation. Dotted line indicates limit of detection (LOD) = 66.67 PFU/ml. Each data point corresponds to each mouse in the group and the number of data points represents the number of mice in the corresponding groups. Source data are provided as a Source Data file.

**Fig. 3:**
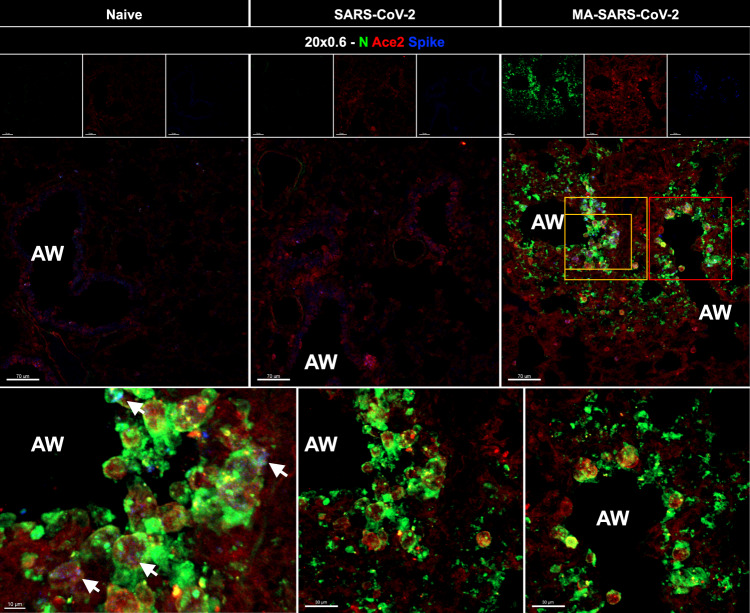
Multiplex immunofluorescence imaging of lung tissues from naïve, WT-SARS-CoV-2, or MA-SARS-CoV-2-infected 129S1 mice immunostained for SARS-CoV-2 N protein (green), ACE2 (red), and SARS-CoV-2 Spike protein (blue). AW: airway. The orange (left and center) and red (right) box is magnified showing N-Protein and Ace2 staining. White arrowheads indicate Ace2+/SARS-CoV-2 N+/SARS-CoV-2 S+ cells. Scale bar: 70, 30, and 10 μm as indicated in the figure panels. *N* = 4 mice per group and one representative image per group is shown.

**Fig. 4:**
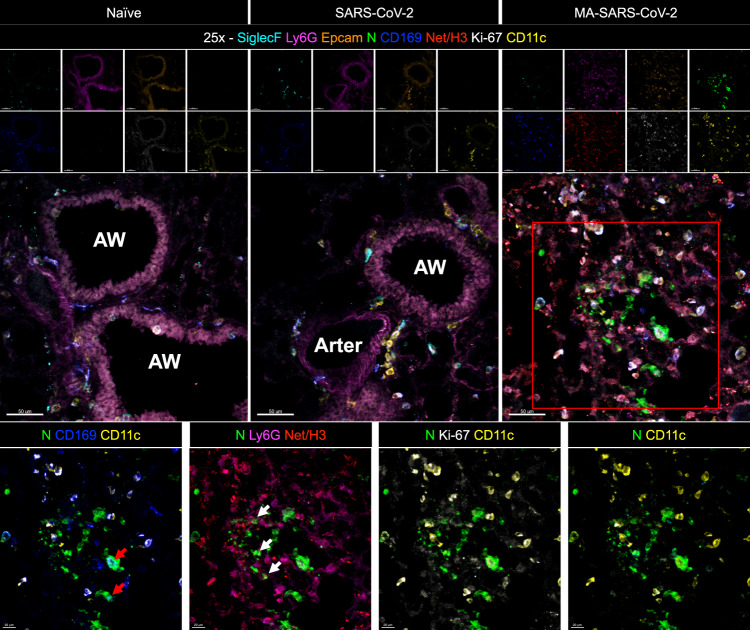
Multiplex immunofluorescence imaging of lung tissues from naïve, SARS-CoV-2, or MA-SARS-CoV-2-infected 129S1 mice at 3 days post-infection immunostained for Siglec-F (teal), Ly6G (magenta), Epcam (orange), SARS-CoV-2 N protein (green), CD169 (blue), Net/H3 (red), Ki-67 (white), and CD11c (yellow). (Left to right) The red box is magnified showing SARS-CoV-2 N-Protein with CD169 + AM, Neutrophils, Ki-67+ AM, and Siglec-F + AM. White arrowheads indicate SARS-CoV-2+/Ly6G+/Net/H3+ cells, red arrowheads indicate SARS-CoV-2+/CD169+/CD11c+ cells. AW: airway. Arter: arteria. Scale bar: 50 μm and 20 μm. *N* = 4 mice per group and one representative image per group is shown.

The pathogenicity of MA-SARS-CoV-2 was examined in terms of its infection and replication potential in laboratory mouse strains of different genetic backgrounds. While the WT-SARS-CoV-2 was unable to infect any of the laboratory strains of mice, MA-SARS-CoV-2 efficiently infected 129S1 (Fig. 1C), C57BL6 (Fig. 1D) as well as BALB/c mice (Fig. 1E) with detectable virus titers in lungs and nasal turbinates of 129S1 mice and lungs of male and female C57BL6 and BALB/c mice, obtained at different days post-infection (DPI) as depicted in Fig. 1. Interestingly, contrary to 129S1 mice, body weight loss upon infection with MA-SARS-CoV-2 was not observed in C57BL/6 or BALB/c mice (not shown). In a repeat experiment MA-SARS-CoV-2 infection of 6–8-week-old 129S1 mice with 2.5 × 10^4^ and 2.5 × 10^5^ PFU resulted in 8% and 11% body weight loss on average by 4 DPI, respectively (Fig. 2A). All mice survived MA-SARS-CoV-2 infection. Virus titers were highest on day 1 in both lungs and nasal turbinates (Fig. 2B). Lung virus titers were close to or below the detection limit by 5DPI, whereas nasal turbinates still had detectable virus titers in all animals at that time point. Pathology was scored on left lung lobes and the scoring system is described in the Methods section and Supplementary Fig. 4. Cumulative pathology scores for individual mice at different time points are given in Fig. 2C. Pathology was mild overall and cumulative pathology scores peaked at 1DPI. Perivascular inflammation was mild and detectable in all animals by 7DPI (Supplementary Fig. 4A). Mild bronchial and peribronchial inflammation was detectable in all animals by 5DPI, whereas alveolar inflammation was not observed in all animals (Supplementary Fig. 4B, C). Finally, epithelial degeneration and necrosis were transient and peaked at 1DPI after which they went back to control levels by 10DPI (Supplementary Fig. 4D). By qPCR, we were able to detect genomic RNA in different tissues at different times post-infection (Fig. 2D). However, we did not observe the presence of replicating virus by plaque assay in tissues other than the lungs. Using multiplex fluorescence microscopy as described before in mouse lung^22^, we confirmed virus infection in pulmonary ACE2 + cells close to airways by staining for both SARS-CoV-2 N and S protein (Fig. 3). When focusing on phagocytes in the infected lung (Fig. 4), we observed that infection with MA-SARS-CoV-2 resulted in neutrophil (Ly6G) attraction to lungs, with the formation of neutrophil extracellular traps (Net/H3 staining). Viral antigen (N protein staining) is found in several phagocyte subsets, including neutrophils (Ly6G+, Net/H3+) and CD169+ alveolar macrophages (AM) (CD11c+, SiglecF+, CD169+). Based on staining for the proliferation marker Ki-67, replicating AM was observed in MA-SARS-CoV-2-infected animals.

### SARS-CoV-2 VoCs harboring the N501Y mutation can directly infect 129S1 mice

**Fig. 5:**
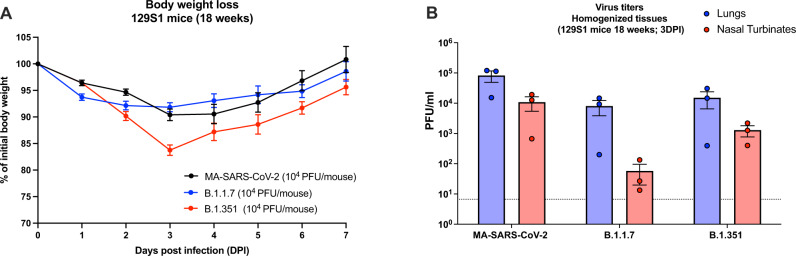
Direct infection of 129S1 mice with VOCs that harbor the N501Y mutation. **A** 18-week-old 129S1 mice were infected with 10^4^ PFU of MA-SARS-CoV-2, B.1.1.7, or B.1.351 (*n* = 10/group). **B** Whole lungs and nasal turbinates were harvested from *n* = 3/group at 3 DPI for virus titration by plaque assay. Bars represent means; error bars represent SEM. Dotted line indicates limit of detection (LOD) = 66.67 PFU/ml. Source data are provided as a Source Data file.

Since the acquisition of the N501Y mutation in the RBD of SARS-CoV-2 spike is suggested to broaden the receptor specificity to murine ACE2, we investigated if two VoCs that have acquired this mutation in nature (B.1.1.7 and B.1.351) could also infect mice directly, similar to the MA-SARS-CoV-2. When 10^4^ PFU was instilled intranasally in 18-week-old 129S1 mice, B.1.1.7 gave similar weight loss compared to MA-SARS-CoV-2, whereas B.1.351 resulted in more pronounced morbidity by 3 DPI (Fig. 5A). Viral titers in lungs and nasal turbinates quantitated by plaque assay were highest for mice infected with MA-SARS-CoV-2 and lowest in B.1.1.7, which was most pronounced in nasal turbinates (Fig. 5B).

### MA-SARS-CoV-2 results in enhanced morbidity in mouse models of obesity, obesity-associated diabetes, and advanced age

**Fig. 6:**
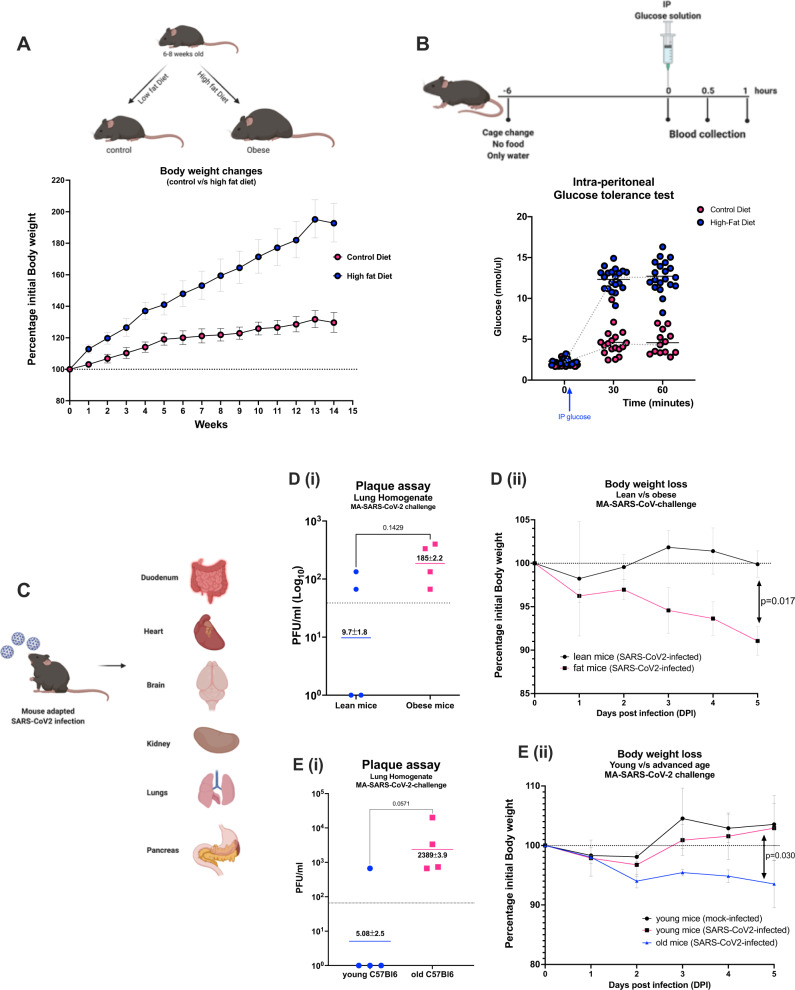
Age and obesity as risk factors for severity associated with MA-SARS-CoV-2. **A** Six- to eight-week-old C57Bl6 mice were fed with sucrose-matched high fat (*n* = 30) or control diet (*n* = 30) for up to 14 weeks and body weight changes were recorded. **B** Mice on control or high-fat diet were fasted for 6 h followed by an intraperitoneal injection of dextrose solution at 2 g/kg body weight. Blood was drawn at different time points by submandibular bleed and blood glucose levels were determined by Glucose assay. **C** Diagrammatic representation of intranasal infection with 1.7 × 10^3^ PFU/mouse of SARS-CoV-2 variant (MA-SARS-CoV-2) followed by harvest of various organs to assess virus replication. **D**, **E** the body weight changes were monitored post-infection until the harvest (*n* = 4–5). Higher lung virus titers were observed in obese mice (**D**-**i**) accompanied with noticeable loss in body weight (**D**-**ii)** as compared to lean/control diet mice. Similarly, the lung virus titers (right lung lobes) (**E**-**i**) and body weight loss (**E**-**ii**) were found to be higher in 52-week-old mice as compared to 6–8-weeks young/control mice, 5 days post-infection. No virus titers were found in other organs harvested 5 days post-infection in both experiments. Two-sided Mann–Whitney U test was performed to calculate the statistical significance between different groups. Symbols represent geometric means; error bars represent standard deviation. Each data point corresponds to individual mouse and the number of data points represents the number of mice in the corresponding groups. Source data are provided as a Source Data file.

The successful establishment of a MA-SARS-CoV-2 allowed us to study risk factors previously associated with severe COVID-19 in humans. We focused on mouse models for obesity, diabetes, and advanced age. Briefly, 6–8-week-old female C57BL6 mice were fed with either a control/low-fat diet (CD) or high-fat diet (HFD) for 14 weeks and the body weight changes were recorded over time. The mice on HFD gained almost twice the original weight and became obese (hence called obese mice) over time as compared to the mice on CD (also referred to as lean mice) (Fig. 6A). The pre-diabetic status of the mice was determined by the intraperitoneal glucose tolerance test^23^ which suggested that the mice on HFD were pre-diabetic while the mice on CD were not (Fig. 6B). The CD and HFD mice were then challenged with a low dose of MA-SARS-CoV-2 (1.7 × 10^3^ PFU/mice) and various organs including the duodenum, heart, brain, kidney, lungs, and pancreas were harvested 5 days post-infection (Fig. 6C). We wanted to test if comorbidities result in more virus-induced morbidity upon infection. We chose to give a lower infection dose as compared to what was used in the initial in vivo titration experiments described above as we assumed that this would provide a larger experimental window. Upon infection, the obese mice showed higher morbidity, as reflected in higher body weight loss over 5 days post-infection as well as higher lung viral titers when compared to the lean mice (Fig. 6D-I, D-ii). We also tested our MA-SARS-CoV-2 on 6–8-week-old and 52-week-old female C57BL6 mice, also referred to as young and old mice, respectively. With the same low viral challenge dose (1.7 × 10^3^ PFU/mice), the 52-week-old mice showed higher morbidity as reflected by body weight loss over 5 days post-infection when compared to young mice (Fig. 6E-i, E-ii). The 52-week-old mice also showed physical symptoms of distress including hunched back and difficulty and reluctance in movement while the 6–8-week-old mice resumed normal activity. Additionally, the viral lung titers were also found to be higher in 52-week-old mice as compared to 6–8-week-old mice at 5 days post-infection. Our data summarizes that both obesity/diabetes and advanced age in mice result in higher morbidity during SARS-CoV-2 infection. Although we did not find detectable virus titers by plaque assay in other organs than the lung with low virus challenge in obese or aged mice, this does not exclude possible extrapulmonary viral replication at other time points or with a higher dose of virus challenge.

### Advanced age results in upregulation of blood cytokine/chemokine levels that are enhanced upon MA-SARS-CoV-2 infection

**Fig. 7:**
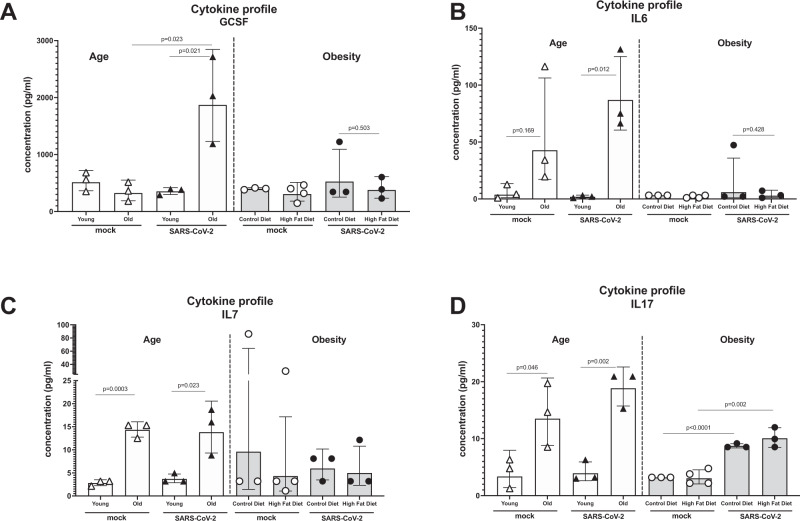
Cytokine profile in blood of comorbid mice. Young (6–8 weeks), old (52 weeks), lean, and obese C57bl/6 mice were infected with 1700 PFU of MA-SARS-CoV-2. Blood was collected at 5 days post-infection (*n* = 3) and cytokine levels were quantified by luminex multiplex ELISA. Levels for GCSF (**A**), IL6 (**B**), IL7 (**C**) and IL17 (**D**) are shown. Two-sided Unpaired *t*-test was performed to calculate the statistical significance between different groups. Bars represent geometric means; error bars represent standard deviation. Each data point corresponds to individual mouse and the number of data points represents the number of mice in the corresponding groups. Source data are provided as a Source Data file.

We performed 32-plex cytokine/chemokine ELISA to identify changes in blood levels of cytokines and chemokines upon MA-SARS-CoV-2 infection in animals with comorbidities at 5 days post-infection. Differences in cytokine levels were mainly observed for GCSF, IL6, IL7, and IL17 (Fig. 7). The observed concentrations for all individual cytokines and chemokines tested are given in supplementary Fig. 5. Enhanced cytokine/chemokine levels were mainly observed in animals with advanced age, whereas the limited sample numbers did not allow us to identify major differences in the blood due to obesity (Fig. 7 and Supplementary Fig. 5). GCSF was enhanced only in aged mice upon infection. IL7 and IL17 were elevated at baseline in aged mice compared to controls, which did not further enhance after infection. IL17 was increased upon infection in lean and obese mice, but not in young mice (Fig. 7D), which may be due to the age difference (young mice: 6–8 weeks, lean and obese mice: 17–19 weeks). IL6 was also elevated at baseline in aged mice compared to controls but did further increase upon infection.

### N501Y and H655Y mutations do not affect SARS-CoV-2 neutralization by human convalescent and post-vaccination serum

**Fig. 8:**
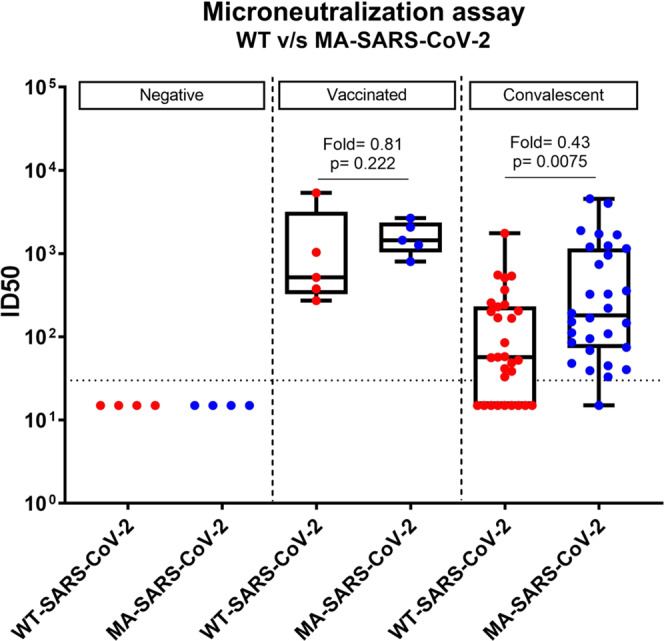
Post-vaccination or convalescent human sera neutralize both WT- and N501Y/H655Y MA-SARS-CoV-2 viruses. Post-vaccination or post-infection human sera were analyzed for virus neutralization by in vitro microneutralization assays using 450TCID_50_ of either WT-SARS-CoV-2 (USA-WA1/2020) or MA-SARS-CoV-2 and the ID50 (inhibition) values were calculated and compared. Each symbol represents ID50 value calculated for a human serum sample [negative (*n* = 4) versus weak (*n* = 8), moderate (*n* = 10), or strong positive (*n* = 11)].Sera from sero-negative individuals were used as negative control for the experiment. Dotted line (LOD). Two-sided Mann–Whitney U test was performed to calculate the statistical significance (p) between different groups. The box-whisker plots show all minimum to maximum data points, with respective medians and interquartile range. Source data are provided as a Source Data file.

For a vaccination study that we recently published^24^ we found that the spike mutations in MA-SARS-CoV-2 do not affect SARS-CoV-2 neutralization by mouse convalescent and post-vaccination serum using in vivo microneutralization assays. To further validate these observations, we performed similar microneutralization assays using sera from study participants with or without SARS-CoV-2 immune responses. We included samples from individuals who had recovered from natural infection or had received the Pfizer/BioNTech SARS-CoV-2 vaccine-BNT162b2 (see Supplementary Table 1). Sera were tested for neutralizing potential against both WT-SARS-CoV-2 (USA-WA1/2020) as well as MA-SARS-CoV-2 strains. Both post-vaccination sera, as well as convalescent sera, neutralize both strains of the virus. Post-vaccination sera had neutralizing antibody titers that were similar to the highest neutralization titers observed in convalescent sera (Fig. 8). Moreover, microneutralization titers against both WT- and MA-SARS-CoV-2 were comparable, if not higher than WT-SARS-CoV-2. Overall, our study shows that the N501Y and H655Y mutations in the SARS-CoV-2 spike protein do not compromise the neutralization potential of this virus by convalescent and post-vaccination human sera.

### Vaccination reduces VoCs escape from neutralization by human convalescent and post-vaccination serum

**Fig. 9:**
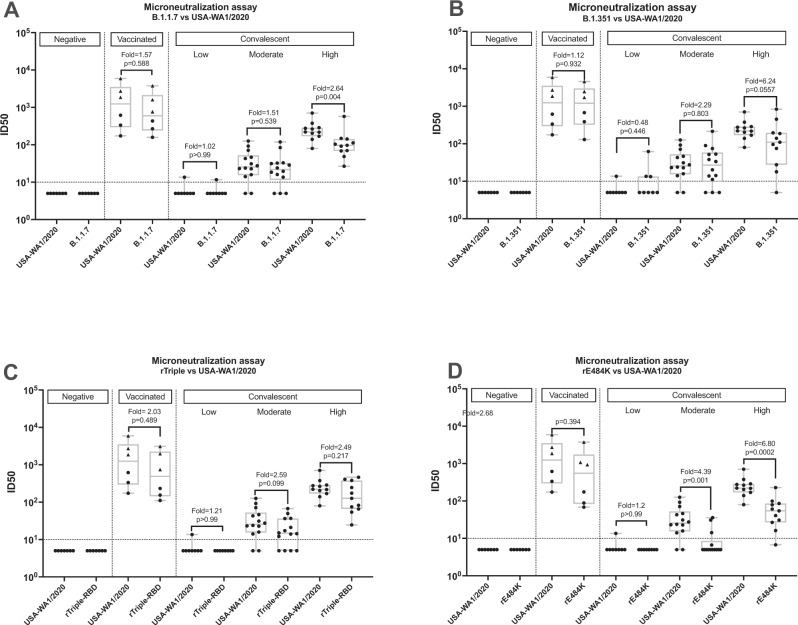
Comparison of in vitro serum microneutralization titers between WT-SARS-CoV-2 (USA-WA1/2020) and B.1.1.7, B.1.351, rTriple- and rE484K-SARS-CoV-2. Box and whisker plots represent median and range of low-to-high value points in each group. The samples are divided into three groups- negative, vaccinated, and convalescent depending on their vaccination status or previous exposure to SARS-CoV-2 [negative (*n* = 7) versus weak (*n* = 8), moderate (*n* = 13), and strong positive (*n* = 18)]. Negative group corresponds to individuals with naïve serum in terms of vaccination or SARS-CoV-2 exposure. Vaccinated samples belong to individuals who received two doses of Pfizer/BioNTech vaccine irrespective of previous exposure to SARS-CoV-2 (convalescent and corresponding convalescent+vaccinated individuals are represented as ▴). Convalescent group is further subdivided into low, moderate and high based on their Spike-specific IgG titres. ID50 values for USA-WA1/2020 are compared to those for B.1.1.7 (**A**), B.1.351 (**B**), rTriple (**C**) and rE484K (**D**). Two-sided Mann–Whitney U tests were performed to calculate statistical differences. The box-whisker plots show all minimum to maximum data points, with respective medians and interquartile range. The fold change was calculated individually for each sample and represented as average fold change for each group. Dotted line (LOD). Source data are provided as a Source Data file.

Next, we performed in vitro microneutralization assays to address the impact of other spike mutations than N501Y on escape from neutralization by human convalescent and post-vaccination sera. Hereto, we used clinical isolates (B.1.1.7 and B.1.351) and recombinant (r)SARS-CoV-2 viruses that harbor either the E484K (rE484K) or K417N + E484K + N501Y (rTriple) mutations. An overview of mutations observed in the viruses used for this study is given in Table 1. For this microneutralization experiment, we included samples from individuals who had recovered from natural infection, received the Pfizer/BioNTech SARS-CoV-2 vaccine-BNT162b2, or both. Post-vaccination sera were obtained from six individuals who received two doses of the Pfizer/BioNTech SARS-CoV-2 vaccine-BNT162b2, three of which had previous exposure to SARS-CoV-2 (see Supplementary Table 2). Sera that were ranked negative or weak for S ELISA titer was also weak or negative for neutralization titers against all viruses tested. Compared to USA-WA1/2020 strain, the neutralization efficiency of strong positive convalescent serum was 2.64- and 6.24-fold lower against B.1.1.7 and B.1.351 strains, respectively (Fig. 9). Similarly, the neutralization titer against rTriple was 2.49-fold lower in the strong positive convalescent group. Reduction in neutralization efficiency was most pronounced with rE484K virus: 6.80-fold. The low and moderate convalescent groups showed low neutralization titers against USA-WA1/2020 which was further reduced or even abolished when tested with B.1.1.7, B.1.351, or the recombinant viruses. Vaccination resulted in neutralization titers against all viruses tested, at comparable levels to the strong positive convalescent group. Importantly, vaccination of convalescent individuals resulted in superior neutralization that was less affected by antigenic changes in the variant or recombinant viruses (Fig. 9 and Supplementary Fig. 6). Surprisingly, the drop in neutralization of B.1.351 or rTriple by convalescent sera was not significant, whereas it was significant for rE484K. Both B.1.351 and rTriple also have the E484K mutation and this discrepancy might be due to the higher variation in neutralization titers observed for B.1.351 and rTriple compared to rE484K.

**Table 1:** Mutations in SARS-CoV-2 variants and r-SARS-CoV-2 used in this study.

Virus	Mutations in Spike
B.1.1.7	N501Y, A570D, D614G, P681H, T716I, S982A, D1118H, HV 69-70 del, Y144 del
B.1.351	D80A, D215G, L243-245del, K417N^a^, E484K, N501Y, D614G, A701V, L242H, L18F
rTriple	N501Y, E484K, K417N
rE484K	E484K

^a^A mixed population of K and N was observed at position 417 for this virus stock, 60% of which was K417N.

## Discussion

There is an urgent need for animal models to test the efficacies of antiviral interventions and to study underlying risk factors associated with enhanced illness during COVID-19. We adapted the USA-WA1/2020 strain to mice by serial passaging the virus in mouse lungs. This resulted in the acquisition of mutations that allow the virus to efficiently replicate to detectable titers in the lungs of mice. Mouse adaptation did not impact growth kinetics of the virus on Vero-E6 cells, which suggests that mutations associated with mouse adaptation broadened receptor specificity rather than changed receptor specificity from human (or primate) ACE-2 to murine ACE-2. We observed multiple mutations associated with different viral genes. The MA-SARS-CoV-2 has acquired an asparagine to tyrosine mutation at position 501 in the spike RBD (N501Y). The same mutation has been described by Gu et al. for mouse-adaptation of SARS-CoV-2 following a similar strategy with aged BALB/c mice^15^. In silico analysis also predicted that the N501Y mutation would result in enhanced RBD affinity for the mACE-2 receptor^21^. We therefore concluded that this mutation is important for mouse adaptation. In a recent prepublication Kuiper et al. suggest that an aromatic substitution at position 498 or 501 is needed for mouse adaptation^25^. This is confirmed by the observation that the Q498Y and N501Y substitutions allow SARS-CoV-2 to replicate in the lungs of wild-type mice (refs. ^21,26^ and this work). Moreover, the N501Y mutation has also occurred in recent VoCs (B.1.1.7, B.1.351, P.1, B.1.1.529) and we show that VoCs B.1.1.7 and B.1.351, contrary to USA-WA1/2020, can indeed infect mice directly (P.1 was not tested at this point and B.1.1.529 experiments are ongoing). Another mutation that got fixed in the S gene upon serial passaging in mice is H655Y, close to the furin cleavage site. This mutation has also been described for VOCs P.1 and the currently circulating B.1.1.529 (Omicron) and is also selected for in other animal models than mice (hamsters and minks)^27^. In the same preprint, Escalera et al. suggest that H655Y is associated with more efficient transmission in a hamster infection model, probably through enhanced spike cleavage and viral growth. Finally, we also observed a four amino-acid insertion in the S protein after amino-acid residue 215 (KLRS). This insertion has been described in SARS-CoV-2 viruses recovered from deer mice upon experimental infection with the same USA-WA1/2020 strain^28^. Minority variants with this insertion were already present in the original SARS-CoV-2 virus obtained from BEI Resources at low frequency and seems also to be enriched after passaging in Vero-E6 cells (data not shown) and mice (this study). Interestingly, cats have recently been identified as an animal species sensitive to SARS-CoV-2 infection^26^ and SARS-CoV-2 with the KLRS insertion was also enriched in lung and GI tract tissues as well as nasal, oropharyngeal, and rectal swabs from infected cats upon experimental infection (Supplementary Fig. 3). Therefore, it is likely that the observed mutations in the spike of MA-SARS-CoV-2 increase viral replication in multiple hosts, not only in mice. The effect of the presence of the KLRS insertion is currently being investigated. We also observed mutations outside of the spike protein of the MA-SARS-CoV-2. As has been shown for influenza virus and SARS-CoV before, multiple viral gene products may contribute to virulence, either individually or by interaction with each other^29–31^. At this point it is still unclear if the mutations outside of spike protein contribute to increased replication and virulence in mice or if they are passenger mutations. Introduction of the observed mutations in a recombinant virus approach for SARS-CoV-2 infectious clone is needed to investigate the individual or synergistic contributions to replication and virulence of these mutations.

Interestingly, the N501Y mutation in the RBD of the S protein is also found in VoCs (B.1.1.7, B.1.351, P.1, B.1.1.529) that are now circulating worldwide with high transmissibility. The N501Y mutation is the only mutation in the RBD of the S protein of the UK-origin B.1.1.7 strains, and therefore, might impact its antigenicity. Moreover, the H655Y mutation present in MA-SARS-CoV-2, VoCs P.1, and B.1.1.529 and may contribute to escape from neutralizing antibodies^32^. The vaccines that are currently being used have an N at position 501 and an H at position 655. Viral escape from neutralizing antibodies has been shown in vitro using pseudoviruses^33^ and there are strong concerns that monoclonal antibody therapies and vaccines that become currently available may have reduced efficacy against circulating strains with the N501Y and/or H655Y mutation^32^. We showed that convalescent and post-vaccination human sera can still potently neutralize the MA-SARS-CoV-2 variant with N501Y and H655Y mutations in its spike protein. Therefore, these mutations may not have a major contribution to the reduction in neutralization against VoCs reported for humoral immune responses induced by current SARS-CoV-2 vaccines^34–36^. All VOCs share the D614G mutation that appeared early during the pandemic, first in viruses circulating in Europe and afterward also in the US. This mutation is suggested to increase the density of SARS-CoV-2 S protein on virions and to increase virulence^37–40^. D614G has only sporadically been associated with minor escape from neutralizing antibodies in convalescent sera so far^39^ and has even been suggested to enhance susceptibility to neutralization^41^. The modest reduction that we observe for post-vaccination sera (1.57 fold) and convalescent sera (2.64 fold) is in line with a previous report in which recombinant SARS-CoV-2 harboring D614G resulted in a similar drop in neutralization efficiency for BNT162b2 vaccination sera^42^. Mutations at position 484 in the S protein (E484K in P.1 and B.1.351, E484Q in B.1.617, E484A in B.1.1.529) have been reported to escape antibody neutralization^43–46^. In fact, the largest drop in antibody neutralization was observed for rE484K, which is in line with our previous report^34^ and suggests that E484K indeed contributes substantially to reduced neutralization observed for B.1.351 and rTriple. In addition, B.1.351 also harbors the K417N substitution, which is described to contribute to escape from neutralizing antibodies^47^. However, this did not seem to result in further reduction of neutralization compared to rE484K in the USA-WA1/2020 backbone. Induction of protective immune responses by vaccination is especially important for people with comorbidities that are at risk for severe illness during COVID-19. Comorbidities include advanced age, type 2 diabetes mellitus, and obesity. Obesity was a predictor of mortality among intensive care unit admitted patients with severe COVID-19, and put patients at higher risk for hypoxemia^6^. Diabetes increases mortality among COVID-19 patients^3^. In this work we present two mouse models for studying enhanced illness during the outcome of SARS-CoV-2 infection in the context of these comorbidities with the MA-SARS-CoV-2 that has the N501Y mutation. This mouse model does not rely on transgenic expression or adenoviral transduction of hACE-2. Therefore, it is suitable to study the effect of ACE inhibitors and angiotensin II type I-receptor blockers that are used to treat diabetics and can result in upregulation of hACE-2, thereby potentially sensitizing patients for severe SARS-CoV-2 infection^48^. MA-SARS-CoV-2 was not lethal in young and healthy mice, in which it caused mild pathology and resulted in a similar degree of body weight loss even when tenfold higher inoculum doses were given (Fig. 2A) and further adaptation to mice may therefore be needed to acquire more mutations associated with enhanced virulence in mice. However, even at a lower infection dose (1700 PFU), obesity and advanced age resulted in higher morbidity (as measured by body weight loss) and reduced control of lung virus titers (Fig. 6). IL6, IL7, and IL17 showed high serum levels at baseline in old mice, reflecting low-grade inflammation associated with immunosenescence (Fig. 7). We did not observe such a pro-inflammatory cytokine profile in obese mouse serum at baseline or upon infection. This is probably due to the low sensitivity of our multiplex ELISA approach using the diluted serum. The strong induction of GCSF upon MA-SARS-CoV-2 in old mice will be the subject of further study to see if it correlates with enhanced recruitment of granulocytes from the bone marrow and pulmonary neutrophil influx in our COVID-19 mouse model.

Taken together we present a mouse-adapted SARS-CoV-2 virus, that acquired mutations that are also observed in VoCs but do not contribute to escape from neutralization by human post-challenge and post-vaccination sera and that can be used to study the impact of SARS-CoV-2 in the context of comorbidities. Our study suggests that vaccine-induced immunity results in superior neutralizing antibody responses, even against newly emerging SARS-CoV-2 variants. It remains to be investigated to what extent other vaccine-induced immune mechanisms, like non-neutralizing antibodies and T cell responses, contribute to protection against new variants, and preclinical animal models like the one presented in this manuscript are indispensable for this research. The emergence of these VoC is a constant reminder that there is a high need for continuous surveillance and rapid vaccine deployment strategies worldwide to deal with this and future pandemics. To prevent and control future outbreaks and excess mortality worldwide, it is inevitable that the majority of the human population is vaccinated as early as possible.

## Methods

This study complies with all relevant ethical regulations which are defined in the respective sections below.

### Reagents

All chemicals for synthesis were purchased from Thermofisher, unless noted otherwise. Horseradish peroxidase (HRP)-conjugated anti-mouse IgG antibody was purchased from Abcam (ab6823). Anti-mouse SARS-CoV-2 N (NP1C7C7) and anti-mouse SARS-CoV-2 S (2BCE5) antibodies were obtained from Center for Therapeutic Antibody Development at Icahn School of Medicine at Mount Sinai, New York. Antibodies used in western blot were anti-mouse-ACE-2 (R&D, MAB3437), anti-beta-tubulin (Sigma–Aldrich, T8328), and anti-mouse secondary antibody (HRP-conjugated, KwikQuant). All antibodies are used at a 1:100 dilution, except for anti-beta-tubulin at 1:1000 dilution.

### Cell lines

Vero-E6 cells (ATTC-CRL 1586, clone E6) are routinely cultured in the laboratory and were maintained in Dulbecco’s Modified Eagle Medium (DMEM; 1× with glucose and sodium pyruvate, Corning 10013CV) supplemented with 10% Fetal Bovine Serum (FBS) from PEAK (PS-FB03), penicillin-streptomycin (100× Corning 30-0002-CL), MEM non-essential amino acids (100× Gibco; 25025CL), and Hepes buffer (1 M, Gibco; 15630-080) at 37 °C with 5% CO_2_. mACE-2 expressing Vero-E6 cells were established by transducing Vero-E6 cells with a lentiviral vector expressing mACE-2 and a puromycin resistance gene (Supplementary Fig. 1). Cells were selected as a polyclonal population by puromycin selection and mACE-2 expression was confirmed by western blot.

### Mouse models for comorbidities

All mice strains (BALB/c, C57BL/6, and 129S1) were obtained from Jackson Laboratories, (MA, USA) and were housed in a pathogen-free facility at Icahn School of Medicine at Mount Sinai, with food and water ad libitum, adhering to the guidelines from Institutional Animal Care and Use Committee (IACUC) of the Icahn School of Medicine at Mount Sinai. Mice were housed in a vivarium with controlled and monitored ambient temperature and humidity with a 12 h day-night light regime. To establish obese mice models, C57BL/6 mice were fed with control or high-fat diets (Research Diets). Mice body weights were recorded over 14 weeks followed by diabetic profiling by intraperitoneal glucose tolerance test. Briefly, mice were moved to fresh cages and fasted for 6 h with access only to water. Mice were then injected intraperitoneally with dextrose solution at 2 g/kg body weight. Blood from mice was drawn by submandibular bleed at 0, 30, and 60 min of injection. Blood glucose levels were estimated by Glucose Assay Kit (abcam) by extrapolation from a standard curve. These obese mice were challenged with MA-SARS-CoV-2 to study the severity of disease progression. To study age as a risk factor for SARS-CoV-2-linked disease severity, 6–8-weeks or 52-weeks-old C57Bl6 mice were used for the mouse-adapted-SARS-CoV-2 challenge.

### SARS-CoV-2 isolates and mouse adaptation

SARS-CoV-2 isolate USA-WA1/2020 (BEI resources; NR-52281), referred to in this manuscript as WT-SARS-CoV-2, was used to challenge mice intranasally. A variant of the virus (termed MA-SARS-CoV-2**)** was obtained after a series of passaging steps in different backgrounds of laboratory mice as well as mACE-2 expressing Vero-E6 cells. Briefly, the virus was serially passaged every 2 days via intranasal inoculation of the virus in 50 ul volume derived from the spun-down supernatants of lung homogenates. The mouse adaptation of the SARS-CoV-2 variant was studied in C57BL/6, BALB/c, and 129S1/SVMJ (termed 129S1 for simplicity in the text and figures) mice models. Viral stocks were sequenced after propagation to verify the integrity of the original viral genome. A variant of concern B.1.1.7 was obtained from BEI resources (NR-54000). A variant of concern B.1.351 (hCoV-19/USA/MD-HP01542/2021 JHU strain) was kindly provided by Dr. Andrew Pekosz from Johns Hopkins Bloomberg School of Public Health.

### Deep sequencing of the viral stocks

To sequence the viral stocks a protocol was developed by ARTIC (https://artic.network/ncov-2019) using the primer set version 3 was used. Viral RNA was purified using Viral-RNA kit (mega-Bio-Tek) following the manufacturer's instructions and used as a template to prepare a cDNA. Overlapping amplicons of ~400 bp covering the whole genome were barcoded using the Oxford Nanopore Technologies (ONT) Native Barcoding Expansion kit (EXP-NBD104). Libraries where prepared according to the manufacturer's instructions, loaded on a minION sequencer equipped with a FLO-MIN106D flow cell. The consensus sequence was obtained using DNAstar software (Lasergene).

### Multi-cycle growth curve for WT and MA-SARS-CoV-2

Confluent Vero-E6 cells in 24 well format were infected with a multiplicity of infection (MOI) of 0.001 of either WT or MA-SARS-CoV-2 virus for 45 min, the inoculum was then removed before supplementing with viral growth media (1× Minimal Essential Medium + 2% FBS + 1% penicillin/streptomycin). Each well was considered as one replicate per time point and supernatants were stored at −80 °C. Viral titers were determined by plaque assay for each sample.

### Virus challenge

2.5 × 10^4^ plaque-forming units (PFU) per mice of WT- or MA-SARS-CoV-2 were used for intranasal infection unless specified otherwise under mild ketamine/xylazine sedation (80 mg/kg ketamine with 12.5 mg/kg xylazine). Body weights were recorded every day to assess the morbidity post-infection until organ harvest. The organs were homogenized in 1× phosphate-buffered saline (PBS) and virus titers were determined by plaque assay. Blood for serology or microneutralization assays was collected either by submandibular bleeding technique or terminally by cardiac puncture.

### Plaque assay

Plaque assays were performed to determine viral titers in samples or organs harvested from mice challenged with USA-WA1/2020, MA-SARS-CoV-2, B.1.1.7, and B.1.351 SARS-CoV-2. Briefly, lungs or other organs were harvested from the mice and homogenized in sterile 1× PBS. After brief centrifugation (10,000 × *g* × 5 min), the tissue debris was discarded, and the supernatant was 10-fold serially diluted starting from 1:10 dilution in 1× PBS. Pre-seeded Vero-E6 or mACE-2-Vero-E6 cells (for WT and MA-SARS-CoV-2, respectively) were infected with tissue homogenate for 1 h at room temperature (RT) followed by an overlay of 2% Oxoid agar mixed with 2× MEM supplemented with 0.3% FBS. The cells were incubated for 72 h at 37 °C and 5% CO_2_ followed by fixation in 1 ml of 10% methanol-free formaldehyde. The plaques were immune-stained with anti-mouse SARS-CoV-2-N (1C7C7; Creative-Biolabs) an antibody for 1 h at RT and consequently with HRP-conjugated anti-mouse secondary IgG antibody for 1 h at room temperature (RT). Finally, the plaques were developed with TrueBlue substrate (KPL-Seracare), and viral titers were calculated and expressed as plaque-forming units per ml (PFU/ml). All antibodies are used at a 1:100 dilution.

### Histopathology

On the day of necropsy, left lung lobes were inflated with 4% formaldehyde in PBS and fixed for 6 days in 4% formaldehyde. Fixed lungs were embedded in paraffin blocks and 5μm sections were cut on a microtome (Microm, Thermo Scientific). Sections were stained with hematoxylin and eosin (H&E) by the Biorepository and Pathology Core (Icahn School of Medicine at Mount Sinai). Sections were mounted using Histomount Solution (Life Technologies) and provided to a veterinary pathologist at the Center for Comparative Medicine and Surgery (CCMS) Comparative Pathology Laboratory, ISMMS (*Dr. Virginia Gillespie*). The pathologist was blinded to the treatment groups. Lung H&Es were evaluated using a pathological scoring system to assess nine parameters: the amount of lung affected, perivascular inflammation, epithelial degeneration/necrosis of bronchi/bronchioles, bronchial/bronchiolar inflammation, and intraluminal debris in bronchi/bronchioles, as well as alveolar inflammation, necrosis in alveoli, fibrin deposition and Type 2 pneumocyte hyperplasia. For area affected a ranking of 0–4 was used where 0 = not affected, 1 = 25%, 2 = 25–50%, 3 = 50–75%, and 4 = 100% of lung affected was used. For histological parameters a score of 1 indicated mild, 2 = moderate, 3 = marked, and 4 = severe.

### Multiplex ELISA

Multiplex ELISA (Millipore) was performed for simultaneous measurements of different cytokines in serum samples from SARS-CoV-2-infected and uninfected mice. The following cytokines were measured: Granulocyte colony-stimulating factor (GCSF), Interleukin (IL)1α, IL2, IL3, IL4, IL6, IL7, IL9, Leukemia inhibitory factor (LIF), IL15, IL17, Macrophage inflammatory protein (MIP1α, MIP1β), Eotaxin, IL1β, IL5, IL10, IL13, Interferon gamma inducible protein (IP10), Macrophage colony-stimulating factor (MCSF), Granulocyte-macrophage colony-stimulating factor (GMCSF), IL12p40, IL12p70, Lipopolysaccharide-induced CXC cytokine (LIX/CXCL5), Keratinocyte derived cytokine (KC), Interferon-gamma (IFN-ϒ), Tumor necrosis factor (TNFα), Vascular endothelial growth factor (VEGF), Regulated on Activation Normal T Cell Expressed and Secreted cytokine (RANTES), Monokine-induced by interferon gamma (MIG), Macrophage inflammatory protein (MIP2), and Monocyte Chemoattractant Protein-1 (MCP1). The assays were performed according to the manufacturer’s instructions and the results were represented as concentrations for each sample in each graph.

### Western blot

Cells were lysed in RIPA buffer (Sigma–Aldrich, USA) supplemented with a protease inhibitor cocktail (Roche, Switzerland). Total protein concentration was determined in each sample by BCA assay and normalized. The lysates were run on a 4–20% gradient polyacrylamide gel at 60 V and transferred onto polyvinylidene fluoride (PVDF) membranes (BioRad Laboratories) using BIO-RAD semi-dry transfer system. PVDF membranes were blocked in 5% non-fat dry milk-containing Tris-buffered saline and 0.1% Tween-20 (TBST). Anti-Tubulin (R&D Systems, Cat# MAB9344) and anti-mACE-2 (R&D Systems, Cat# MAB3437) primary antibodies were used at a dilution of 1:1000 while secondary HRP-conjugated antibodies were used at dilutions of 1:10,000 in 3% BSA-containing TBST.

### Lung tissue preparation for confocal microscopy

Lung tissues were processed as previously described^22^. Briefly, tissues were fixed in paraformaldehyde overnight at 4 °C followed by 30% sucrose overnight at 4 °C and subsequent embedding in OCT media. 20 um frozen tissue sections were sectioned using Leica CM3050S cryostat. FcRs were blocked with anti-CD16/32 Fc block antibody (Clone 93, Biolegend) diluted in 1× PBS containing 2% serum, (2% fetal bovine serum FBS), and 0.1% Triton-X for 1 h at room temperature. Sections were stained with the antibodies anti-Siglec-F (Clone BE50-2440, BD Bioscience), anti-Ly6G (Clone 1A8, BD Bioscience), anti-EpCAM (Clone G8.8, Biolegend), anti-N (1C7C7), anti-S (2BCE5), anti-Neutrophil Elastase (polyclonal, abcam), anti-H3 (polyclonal, abcam), Ki-67 (Clone 16A8, Biolegend), CD11c (Clone N418, Invitrogen), anti-ACE2 (polyclonal, abcam), and CD169 (Clone Ser-4, Invitrogen) that were diluted in PBS containing 2% serum, 2% FBS, and 0.1% Triton-X for 1 h at room temperature. Followed by 3 washes with 1× PBS and stained with secondary Goat anti-Rabbit 555 (Invitrogen) antibody diluted in 1× PBS containing 2% serum, 2% FBS, and 0.1% Triton-X for 1 h at room temperature. Images were acquired using a Zeiss LSM 880 confocal microscope (Carl Zeiss) at ×25 magnification with the Zen Black software or a Zeiss LSM800 (Carl Zeiss) at ×20 magnification with the Zen Blue software. The imaging data were processed and analyzed using Imaris software version 9.2.1 (Bitplane; Oxford Instruments). All antibodies were used at a 1:100 dilution.

### 50% tissue culture infective dose (TCID_50_) calculation and in vitro microneutralization assay

To estimate the neutralizing efficiency of sera from vaccinated or SARS-CoV-2-infected mice or humans, in vitro microneutralization assays were performed similarly to what is described previously^49^. Briefly, the mice or human sera were inactivated at 56 °C for 30 min. Serum samples were serially diluted 3-fold starting from 1:10 dilution in Vero-E6-infection medium (DMEM + 2% FBS + 1% non-essential amino acids). The samples were incubated with optimized tissue culture infective dose 50 (TCID_50_), as described in the figure legends, of either WT- or MA-SARS-CoV-2 for 1 h in an incubator at 37 °C, 5% CO_2_ followed by incubation with pre-seeded Vero-E6 at 37 °C for 48 h. The plates were fixed in 4% formaldehyde at 4 °C overnight. For TCID_50_ calculation, the virus stock was serially diluted 10-fold starting with 1:10 dilution and incubated on Vero-E6 cells for 48 h followed by fixation in 4% Formaldehyde. The cells were washed with 1X PBS and permeabilized with 0.1% Triton X-100 in 1× PBS. The cells were washed again and blocked in 5% non-fat milk in 1X PBS + 0.1% Tween-20 for 1 h at room temperature. After blocking, the cells were incubated with anti-SARS-CoV-2 N (1C7C7) and anti-S (2BCE5) monoclonal antibodies, mixed in 1:1 ratio, for 1.5 h at room temperature. Both antibodies were used at a 1:100 dilution. The cells were washed in 1× PBS and incubated with 1:5000 diluted HRP-conjugated anti-mouse IgG secondary antibody for 1 h at RT followed by a brief 1× PBS wash. Finally, 100 μl tetramethyl benzidine (TMB) substrate was added and incubated at RT until blue color appeared, and the reaction was terminated with 50 μl 1 M H_2_SO_4_. Absorbance was recorded at 450 nm and 650 nm and percentage reduction in infection was calculated as compared to the negative control.

### Serum samples from human subjects

#### Ethics statement

The study protocols for the collection of clinical specimens from individuals with and without SARS-CoV-2 infection by the Personalized Virology Initiative were reviewed and approved by the Mount Sinai Hospital Institutional Review Board (IRB-16-00791; IRB-20- 03374). All participants provided informed consent prior to the collection of specimens and clinical information. All specimens were coded prior to processing.

#### Sample collection

The first set of 33 human serum samples was selected from study participants based on their SARS-CoV-2 spike enzyme-linked immunosorbent assay (ELISA) antibody titer (negative [*N* = 4] versus weak [*N* = 8], moderate [*N* = 10], or strong positive [*N* = 11]). In addition, we included sera from six individuals that had received two doses of the Pfizer SARS-CoV-2 vaccine (V1–V6). Demographics and available metadata for each participant is summarized in Supplementary Table 1. A second set of 46 human serum samples was selected in a similar way based on SARS-CoV-2 spike ELISA antibody titers (negative [*n* = 7] versus weak [*n* = 8], moderate [*n* = 13], and strong positive [*n* = 18]. Demographics and metadata for each participant in the second set are summarized in Supplementary Table 2. All serum samples were heat-inactivated (56 °C, 1 h) and all experiments were conducted in a blinded manner.

### Software

Statistics were performed with GraphPad Prism version 8.0.0 for Windows, GraphPad Software, San Diego, California USA, www.graphpad.com. Figures were made with GraphPad Software and BioRender.com.

### Reporting summary

Further information on research design is available in the Nature Research Reporting Summary linked to this article.

## Supplementary information

Supplementary Information

Reporting Summary

## Data Availability

Sequencing data of the MA-SARS-CoV-2 genome are deposited to public repository (GISAID accession # EPI_ISL_12243860). No code was generated for this work. Unique biological materials generated for the work presented in this manuscript are available upon request for research purposes and will be shared according to standard material transfer agreements between research institutes. Requests for a material transfer agreement (MTA) can be addressed to Michael Schotsaert at Michael.Schotsaert@mssm.edu. Source data are provided with this paper.

## Source data

Source Data

## References

[1] Hoffmann M (2020). SARS-CoV-2 cell entry depends on ACE2 and TMPRSS2 and is blocked by a clinically proven protease inhibitor. Cell.

[2] Kruglikov IL, Shah M, Scherer PE (2020). Obesity and diabetes as comorbidities for COVID-19: underlying mechanisms and the role of viral–bacterial interactions. eLife.

[3] Wu Z-H, Tang Y, Cheng Q (2020). Diabetes increases the mortality of patients with COVID-19: a meta-analysis. Acta Diabetol..

[4] Wang B, Li R, Lu Z, Huang Y (2020). Does comorbidity increase the risk of patients with COVID-19: evidence from meta-analysis. Aging.

[5] X. Y (2020). Clinical course and outcomes of critically ill patients with SARS-CoV-2 pneumonia in Wuhan, China: a single-centered, retrospective, observational study. Lancet Respir. Med..

[6] Pettit NN (2020). Obesity is associated with increased risk for mortality among hospitalized patients with COVID‐19. Obesity.

[7] Du R-H (2020). Hospitalization and critical care of 109 decedents with COVID-19 pneumonia in Wuhan, China. Ann. Am. Thorac. Soc..

[8] Zhou P (2020). A pneumonia outbreak associated with a new coronavirus of probable bat origin. Nature.

[9] Rathnasinghe R (2020). Comparison of transgenic and adenovirus hACE2 mouse models for SARS-CoV-2 infection. Emerg. Microbes Infect..

[10] Muñoz-Fontela C (2020). Animal models for COVID-19. Nature.

[11] Hassan AO (2020). A SARS-CoV-2 infection model in mice demonstrates protection by neutralizing antibodies. Cell.

[12] Sun S-H (2020). A mouse model of SARS-CoV-2 infection and pathogenesis. Cell Host Microbe.

[13] Dinnon KH (2020). A mouse-adapted model of SARS-CoV-2 to test COVID-19 countermeasures. Nature.

[14] Leist SR (2020). A mouse-adapted SARS-CoV-2 induces acute lung injury and mortality in standard laboratory mice. Cell.

[15] Gu H (2020). Adaptation of SARS-CoV-2 in BALB/c mice for testing vaccine efficacy. Science.

[16] Wang J (2020). Mouse-adapted SARS-CoV-2 replicates efficiently in the upper and lower respiratory tract of BALB/c and C57BL/6 J mice. Protein Cell.

[17] Davies NG (2021). Estimated transmissibility and impact of SARS-CoV-2 lineage B.1.1.7 in England. Science.

[18] Haddock E, Feldmann H, Marzi A (2018). Ebola virus infection in commonly used laboratory mouse strains. J. Infect. Dis..

[19] Elbahesh H, Schughart K (2016). Genetically diverse CC-founder mouse strains replicate the human influenza gene expression signature. Sci. Rep..

[20] Petkova SB (2008). Genetic influence on immune phenotype revealed strain-specific variations in peripheral blood lineages. Physiol. Genomics.

[21] Rodrigues JPGLM (2020). Insights on cross-species transmission of SARS-CoV-2 from structural modeling. PLoS Comput. Biol..

[22] Ural BB (2020). Identification of a nerve-associated, lung-resident interstitial macrophage subset with distinct localization and immunoregulatory properties. Sci. Immunol..

[23] Ayala JE (2010). Standard operating procedures for describing and performing metabolic tests of glucose homeostasis in mice. Dis. Model. Mech..

[24] Jangra S (2021). A combination adjuvant for the induction of potent antiviral immune responses for a recombinant SARS-CoV-2 protein vaccine. Front. Immunol..

[25] Kuiper, M. J., Wilson, L. O., Mangalaganesh, S., Reti, D. & Vasan, S. S. But Mouse, you are not alone: On some severe acute respiratory syndrome coronavirus 2 variants infecting mice. *ILAR J*. 10.1101/2021.08.04.455042 (2021). 10.1093/ilar/ilab031 PMC9236659 35022734

[26] Shi J (2020). Susceptibility of ferrets, cats, dogs, and other domesticated animals to SARS-coronavirus 2. Science.

[27] Escalera, A. et al. SARS-CoV-2 variants of concern have acquired mutations associated with an increased spike cleavage. *bioRxiv*10.1101/2021.08.05.455290 (2021).

[28] Fagre, A. et al. SARS-CoV-2 infection, neuropathogenesis and transmission among deer mice: implications for reverse zoonosis to New World rodents. *bioRxiv*10.1101/2020.08.07.241810 (2020). 10.1371/journal.ppat.1009585 PMC8168874 34010360

[29] Grimm D (2007). Replication fitness determines high virulence of influenza A virus in mice carrying functional Mx1 resistance gene. Proc. Natl Acad. Sci. USA.

[30] Smeenk CA, Wright KE, Burns BF, Thaker AJ, Brown EG (1996). Mutations in the hemagglutinin and matrix genes of a virulent influenza virus variant, A/FM/1/47-MA, control different stages in pathogenesis. Virus Res..

[31] Roberts A (2007). A mouse-adapted SARS-coronavirus causes disease and mortality in BALB/c mice. PLoS Pathog..

[32] Baum A (2020). Antibody cocktail to SARS-CoV-2 spike protein prevents rapid mutational escape seen with individual antibodies. Science.

[33] Weisblum Y (2020). Escape from neutralizing antibodies by SARS-CoV-2 spike protein variants. eLife.

[34] Jangra S (2021). SARS-CoV-2 spike E484K mutation reduces antibody neutralisation. Lancet Microbe.

[35] Alenquer M (2021). Signatures in SARS-CoV-2 spike protein conferring escape to neutralizing antibodies. PLoS Pathog..

[36] Ikegame S (2021). Neutralizing activity of Sputnik V vaccine sera against SARS-CoV-2 variants. Nat. Commun..

[37] Zhang L (2020). SARS-CoV-2 spike-protein D614G mutation increases virion spike density and infectivity. Nat. Commun..

[38] Korber B (2020). Tracking changes in SARS-CoV-2 Spike: evidence that D614G increases infectivity of the COVID-19 virus. Cell.

[39] Hu, J. et al. The D614G mutation of SARS-CoV-2 spike protein enhances viral infectivity and decreases neutralization sensitivity to individual convalescent sera. *bioRxiv*10.1101/2020.06.20.161323 (2020).

[40] Plante, J. A. et al. Spike mutation D614G alters SARS-CoV-2 fitness and neutralization susceptibility. *bioRxiv*10.1101/2020.09.01.278689 (2020).

[41] Weissman D (2021). D614G Spike mutation increases SARS CoV-2 susceptibility to neutralization. Cell Host Microbe.

[42] Zou J (2021). The effect of SARS-CoV-2 D614G mutation on BNT162b2 vaccine-elicited neutralization. npj Vaccines.

[43] Xie X (2021). Neutralization of SARS-CoV-2 spike 69/70 deletion, E484K and N501Y variants by BNT162b2 vaccine-elicited sera. Nat. Med..

[44] Kim YJ, Jang US, Soh SM, Lee J-Y, Lee H-R (2021). The impact on infectivity and neutralization efficiency of SARS-CoV-2 lineage B.1.351 pseudovirus. Viruses.

[45] Wall EC (2021). Neutralising antibody activity against SARS-CoV-2 VOCs B.1.617.2 and B.1.351 by BNT162b2 vaccination. Lancet Lond. Engl..

[46] Edara V-V (2021). Infection and vaccine-induced neutralizing-antibody responses to the SARS-CoV-2 B.1.617 variants. N. Engl. J. Med..

[47] Yuan M (2021). Structural and functional ramifications of antigenic drift in recent SARS-CoV-2 variants. Science.

[48] Fang L, Karakiulakis G, Roth M (2020). Are patients with hypertension and diabetes mellitus at increased risk for COVID-19 infection?. Lancet Respir. Med..

[49] Amanat F (2020). An in vitro microneutralization assay for SARS‐CoV‐2 serology and drug screening. Curr. Protoc. Microbiol..

