# Supplementary material for: Characterization of SARS-CoV-2 Spike mutations important for infection of mice and escape from human immune sera
Source: Nat Commun. 2022 Jul 7;13:3921. doi: 10.1038/s41467-022-30763-0 (PMC9261898; doi:10.1038/s41467-022-30763-0)
Supplement: Supplementary file 1 — Supplementary Information [file 41467_2022_30763_MOESM1_ESM.pdf]

Supplementary Information: Characterization of SARS-CoV-2 Spike mutations important for infection of mice and escape from human immune sera

Rathnasinghe *et al.*

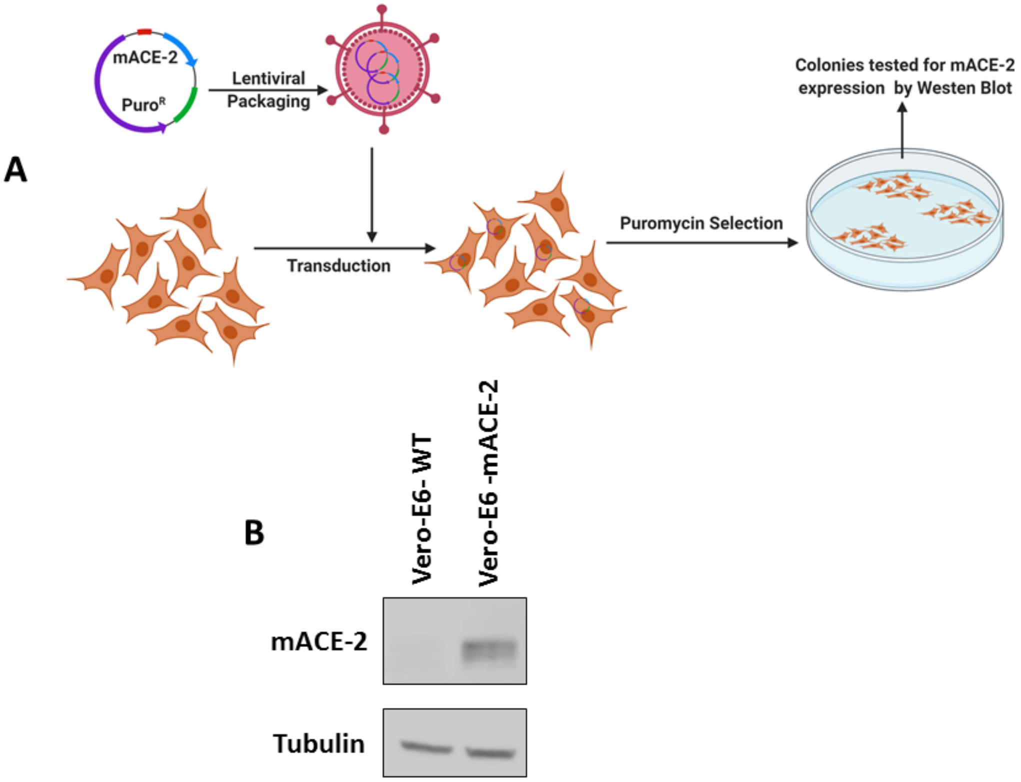

Supplementary Fig. 1. Establishing mACE-2 Vero-E6 cells. (A) Strategy: Vero-E6 cells were transduced with a lentiviral vector expressing mACE-2 and a puromycin resistance gene. Cells were selected for mACE-2 expression by puromycin selection. **(B)** Expression in the selected polyclonal population was confirmed by Western blot. The Western blot experiment was done once.

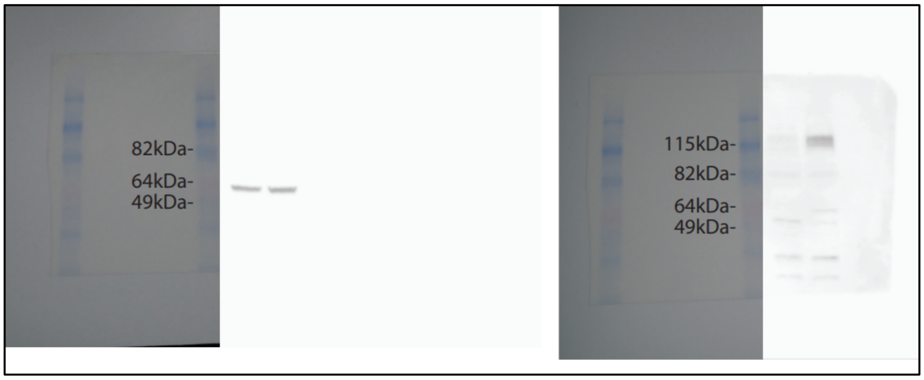

Original Blots (Left: Tubulin; Right: mACE-2)

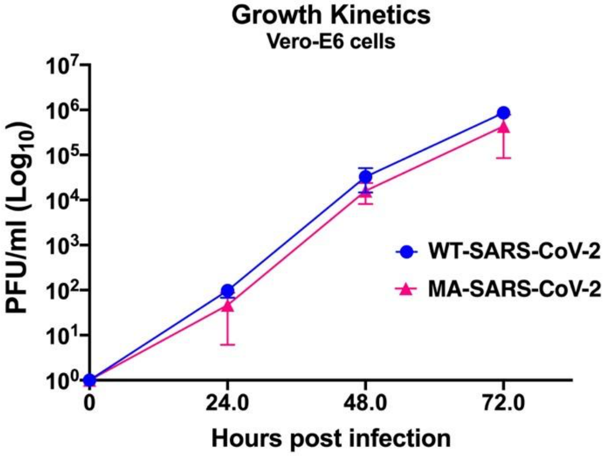

Supplementary Fig. 2. Comparison of growth kinetics of WT-SARS-CoV2 and MA-SARS-CoV-2.

Vero-E6 cells were infected with equal PFUs of WT-SARS-CoV-2 or MA-SARS-CoV-2 and supernatant media was collected at different time points. The virus replication was titrated by plaque assay. No major difference was observed in growth kinetics of WT and MA-SARS-CoV-2 *in-vitro*. Symbols represent means, error bars represent standard error (n=3).

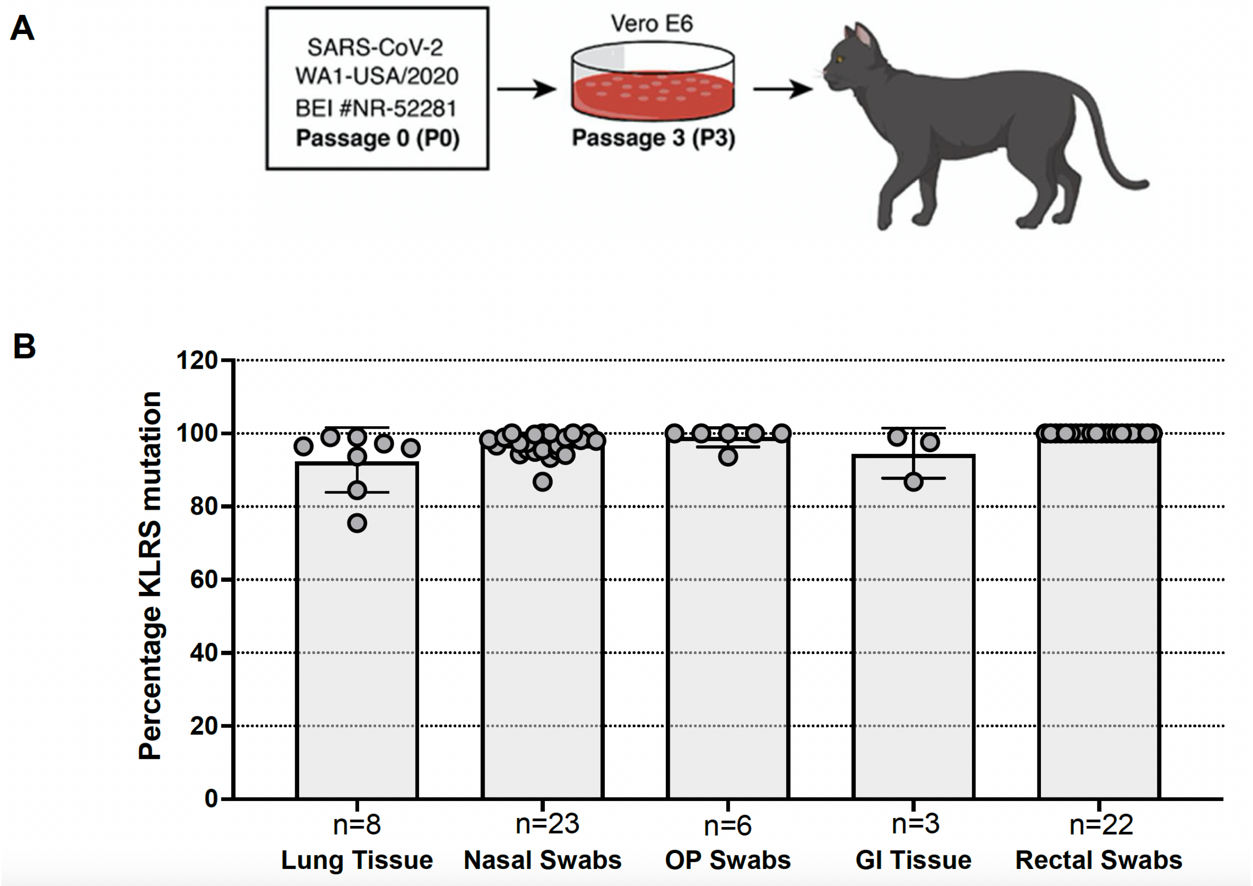

Supplementary Fig. 3: Increased prevalence of KLRS insertion in SARS-CoV-2-infected cats. (A) The SARS-CoV-2 WA1-USA/2020 strain from BEI was passaged three times in Vero E6 cells before being used to infect six cats intranasally and orally with a 10^6 $TCID_{50}$ dose of virus that were subsequently exposed to 2 sentinel contact cats that also became infected. Nasal, oropharyngeal (OP), and rectal swabs were collected from cats on 2 to 7 days post-challenge (DPC) and lung/Gastrointestinal (GI) tissues were collected on 4-7 DPC. RNA was extracted, sequenced, and analyzed to determine the relative percentage of the KLRS insertion in various clinical samples from cats. **(B)** Chart showing that the prevalence of the KLRS insertion increases in cats with 90% - 100% prevalence in various swabs or tissues. The bars and error bars represent geometric mean and geometric SD, respectively.

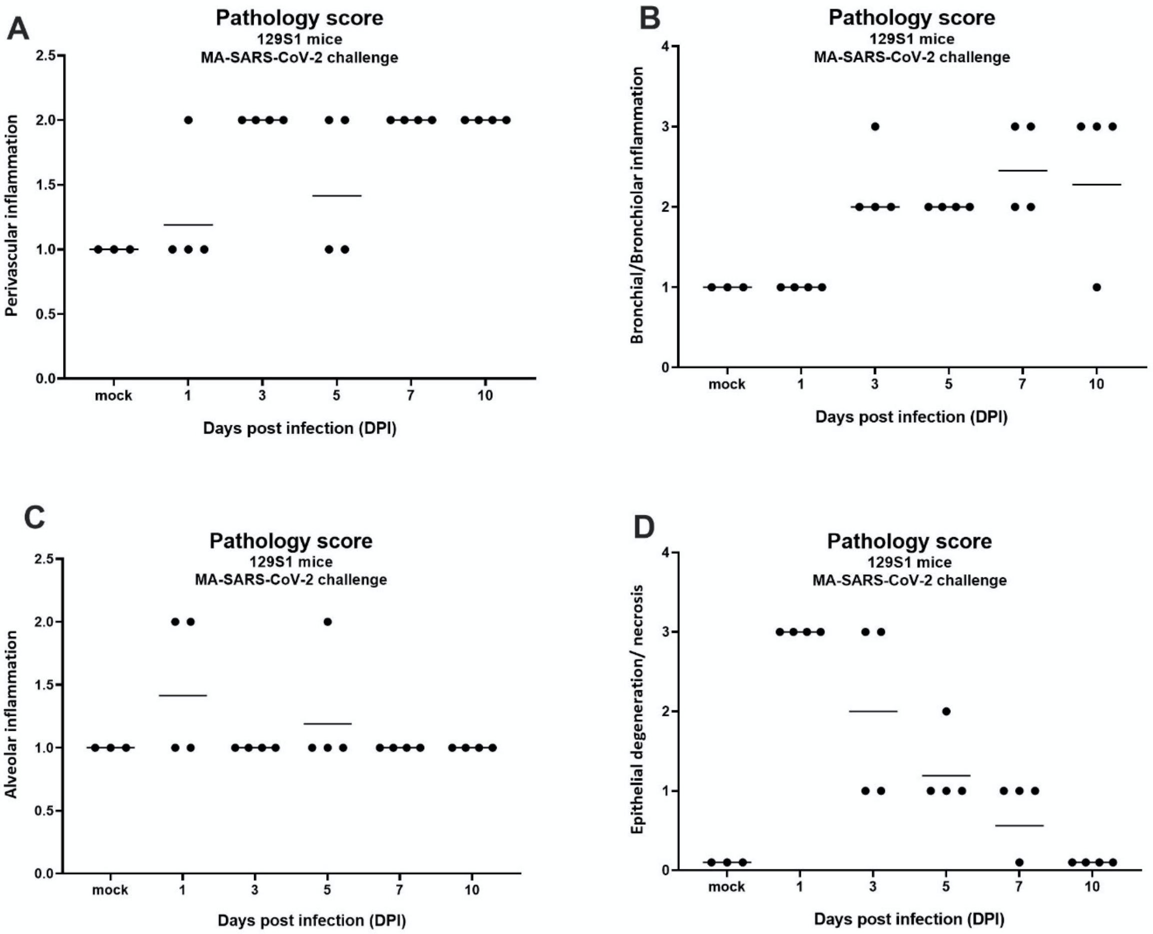

Score	Area affected	Epithelial degeneration/necrosis	Inflammation
0	none	none	None
1	5-10%	Minimal; scattered cell necrosis/vacuolation affecting 5 to 10% of tissue section	Minimal; scattered inflammatory cells affecting 5-10% of tissue section
2	10-25%	Mild; scattered cell necrosis/vacuolation	multifocal, few inflammatory cells
3	25-50%	Moderate; multifocal vacuolation or sloughed/necrotic cells	Thin layer of cells (<5 cell layer thick)
4	50-75%	Marked; multifocal/segmental necrosis, epithelial loss/effacement	Thick layer of cells (>5 cell layer thick)
5	>75%	Severe; coalescing areas of necrosis, parenchymal effacement	Confluent areas of inflammation

Supplementary Fig. 4. Pathology in lungs of 129S1 mice harvested on different days post infection. Individual lung pathology scored during the course of infection with 2.5×10^4 PFU of MA-SARS-CoV-2 (n=3 for mock; n=4 for other groups). Each dot represents individual mice and the bar represents geometric mean of score in respective group.

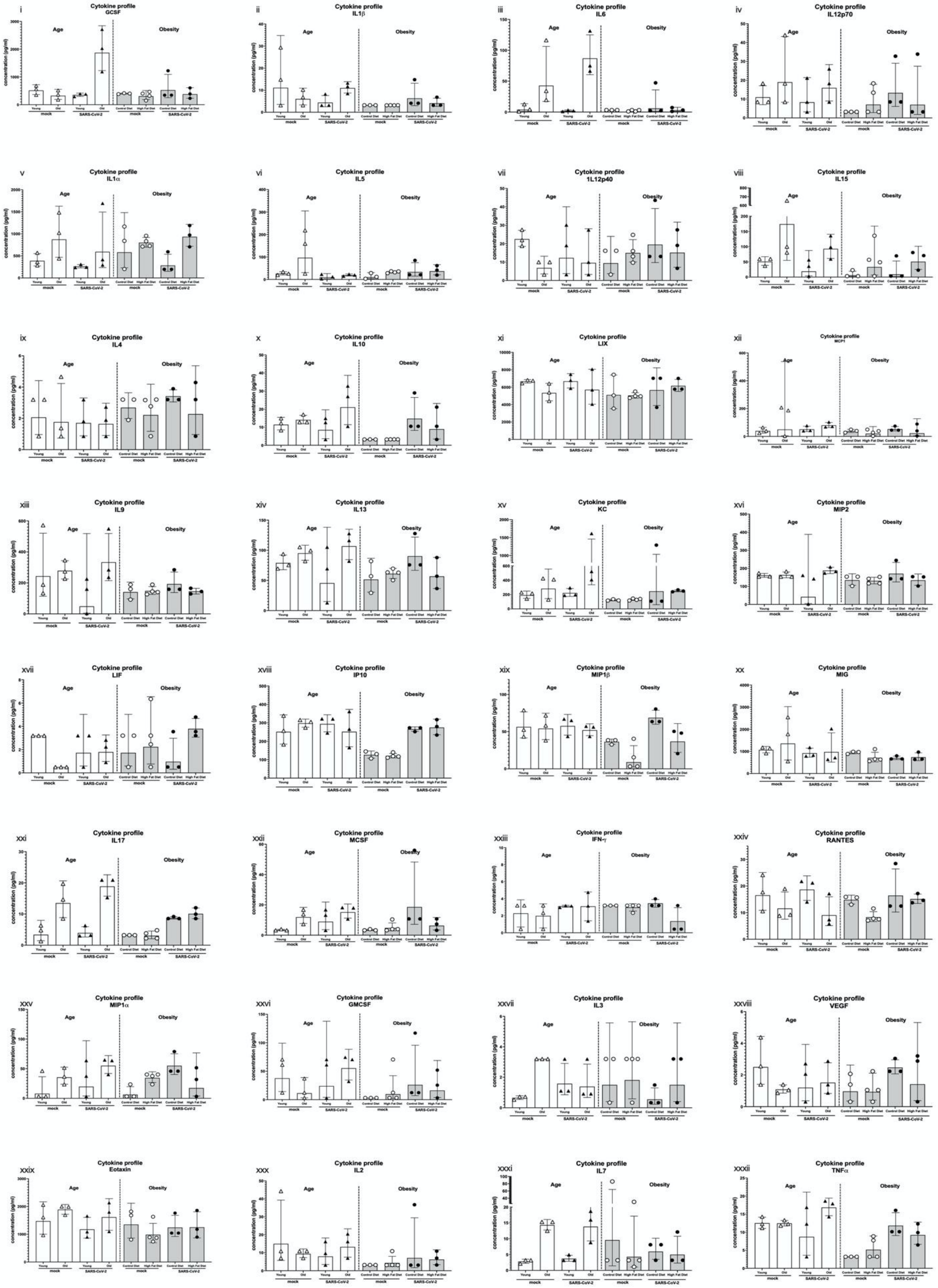

Supplementary Fig. 5. Cytokine profile in blood of comorbid mice. Young (6-8 weeks), old (52 weeks), lean and obese C57bl/6 mice were infected with 1700 PFU of MA-SARS-CoV-2. Blood was collected at 5 days post infection and cytokine levels were quantified by luminex multiplex ELISA (n=4 for High fat diet, mock-challenged group and n=3 for all other groups). Bars represent geometric means; error bars represent standard deviation.

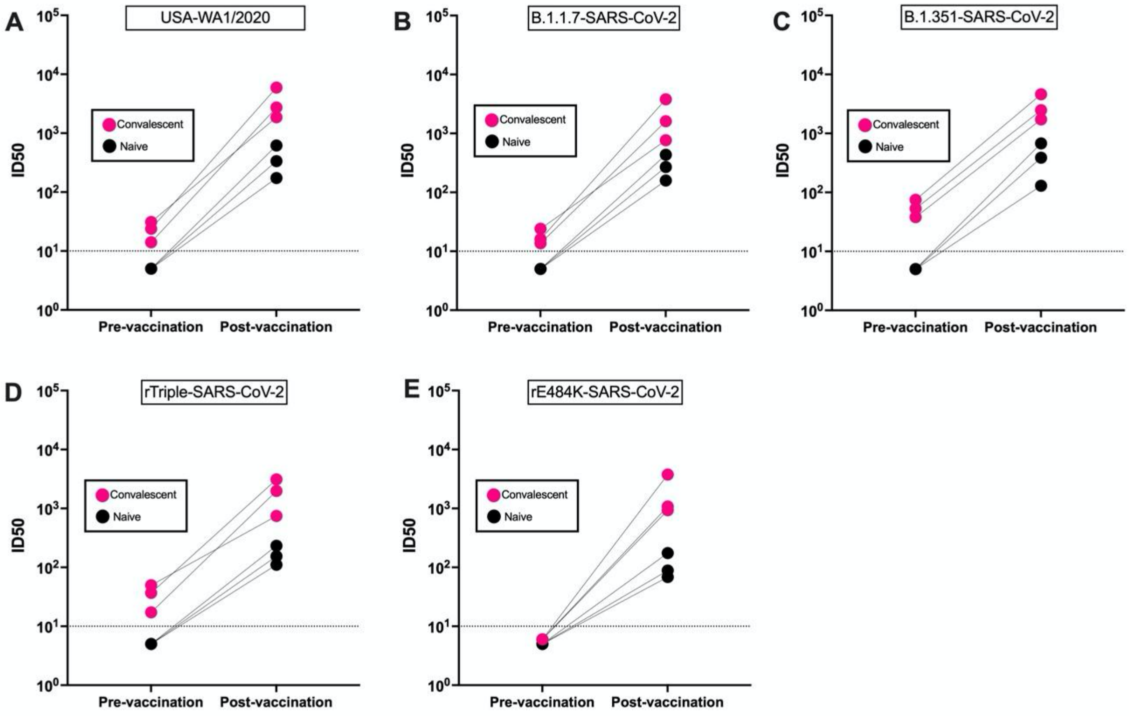

Supplementary Fig. 6. Convalescent sera are boosted to higher neutralization titres after vaccination. Vaccinated individuals who previously contracted SARS-CoV-2 (represented as convalescent) were compared with vaccinated individuals who did not (represented as naive). Pre- and post-vaccination ID50 values were compared for each individual within same virus group- USA-WA1/2020 (A), B.1.1.7 (B), B.1.351 (C), rTriple (D) and rE484K (E).

Supplementary table 1: Description of serum samples obtained from human subjects

SERUM				
Seropositive, vaccine	**Spike IgG response**	**Sex**	**Age group (yrs)**	**Days post 1 vaccine dose (Pfizer)**
V1	Strong positive	F	>60	68
V2	Strong positive	M	30-40	47
V3	Strong positive	F	50-60	47
V4	Strong positive	M	>60	48
V5	Strong positive	F	40-50	49
V6	Strong positive	F	30-40	48
Seropositive, infection	**Spike IgG response**	**Sex**	**Age group (yrs)**	**Days post onset of symptoms**
P1	Weak positive	M	20-29	260
P2	Weak positive	M	50-59	NA
P3	Weak positive	F	30-39	111
P4	Weak positive	F	30-39	221
P5	Weak positive	F	30-39	254
P6	Weak positive	F	20-29	247
P7	Weak positive	M	30-39	220
P8	Weak positive	F	20-29	Asymptomatic
P9	Moderate positive	M	30-39	NA
P10	Moderate positive	F	30-39	197
P11	Moderate positive	F	50-59	Asymptomatic
P12	Moderate positive	F	30-39	Asymptomatic
P13	Moderate positive	F	20-29	273
P14	Moderate positive	M	30-39	Asymptomatic
P15	Moderate positive	F	20-29	258
P16	Moderate positive	F	20-29	246
P17	Moderate positive	M	20-29	Asymptomatic
P18	Moderate positive	F	50-59	204
P19	Strong positive	F	50-59	NA
P20	Strong positive	F	30-39	245
P21	Strong positive	M	NA	170
P22	Strong positive	F	>60	Asymptomatic
P23	Strong positive	F	40-49	NA
P24	Strong positive	F	50-59	191
P25	Strong positive	F	30-39	NA
P26	Strong positive	F	50-59	113
P27	Strong positive	M	>60	Asymptomatic
P28	Strong positive	M	18-19	218
P29	Strong positive	M	50-59	219

Seronegative, post pandemic	**Spike IgG response**	**Sex**	**Age group (yrs)**	**Days from last negative serology**
N1	Negative	F	40-50	23
N2	Negative	F	20-29	24
N3	Negative	F	20-29	23
N4	Negative	F	30-35	22

Supplementary table 2: Description of serum samples obtained from human subjects

SERUM				
Seropositive, vaccine	**Spike IgG response**	**Sex**	**Age group (yrs)**	**Matched pre and post vaccine samples (Pfizer)**
V1	Negative	F	30-39	pre-vaccine
V2	Negative	F	30-39	pre-vaccine
V3	Negative	F	30-39	pre-vaccine
V1	Strong positive	F	30-39	post 2nd vaccine dose
V2	Strong positive	F	30-39	post 2nd vaccine dose
V3	Strong positive	F	30-39	post 2nd vaccine dose
V4	Moderate positive	M	30-39	pre-vaccine
V5	Strong positive	F	30-39	pre-vaccine
V6	Moderate positive	M	40-49	pre-vaccine
V4	Strong positive	M	30-39	post 2nd vaccine dose
V5	Strong positive	F	30-39	post 2nd vaccine dose
V6	Strong positive	M	40-49	post 2nd vaccine dose
Seronegative, post pandemic		**Sex**	**Age group (yrs)**	**Days from last negative serology**
N1	Negative	F	40-50	23
N2	Negative	F	20-29	24
N3	Negative	F	20-29	23
N4	Negative	F	30-35	22
Seropositive, infection		**Sex**	**Age group (yrs)**	**Days post onset of symptoms**
P1	Weak positive	M	20-29	260
P2	Weak positive	M	50-59	NA
P3	Weak positive	F	30-39	111
P4	Weak positive	F	30-39	221
P5	Weak positive	F	30-39	254
P6	Weak positive	F	20-29	247
P7	Weak positive	M	30-39	220
P8	Weak positive	F	20-29	Asymptomatic
P9	Moderate positive	M	30-39	NA
P10	Moderate positive	F	30-39	197
P11	Moderate positive	F	50-59	Asymptomatic
P12	Moderate positive	F	30-39	Asymptomatic
P13	Moderate positive	M	30-39	234

P14	Moderate positive	F	20-29	273
P15	Moderate positive	M	30-39	Asymptomatic
P16	Moderate positive	F	20-29	258
P17	Moderate positive	F	20-29	246
P18	Moderate positive	M	20-29	Asymptomatic
P19	Moderate positive	F	50-59	204
P20	Strong positive	F	50-59	NA
P21	Strong positive	F	30-39	245
P22	Strong positive	M	NA	170
P23	Strong positive	F	>60	Asymptomatic
P24	Strong positive	F	40-49	NA
P25	Strong positive	F	50-59	191
P26	Strong positive	F	30-39	NA
P27	Strong positive	F	50-59	113
P28	Strong positive	M	>60	Asymptomatic
P29	Strong positive	M	18-19	218
P30	Strong positive	M	50-59	219
