# Supplementary material for: Characterization of SARS-CoV-2 Spike mutations important for infection of mice and escape from human immune sera
Source: Nat Commun. 2022 Jul 7;13:3921. doi: 10.1038/s41467-022-30763-0 (PMC9261898; doi:10.1038/s41467-022-30763-0)
Supplement: Supplementary file 2 — Reporting Summary [file 41467_2022_30763_MOESM2_ESM.pdf]

nature research

Corresponding author(s): Michael Schotsaert
Adolfo Garcia-Sastre

Last updated by author(s): 2022/04/24

Reporting Summary

Nature Research wishes to improve the reproducibility of the work that we publish. This form provides structure for consistency and transparency in reporting. For further information on Nature Research policies, see our Editorial Policies and the Editorial Policy Checklist.

Statistics

For all statistical analyses, confirm that the following items are present in the figure legend, table legend, main text, or Methods section.

n/a	Confirmed	
[ ]	[x]	The exact sample size (n) for each experimental group/condition, given as a discrete number and unit of measurement
[x]	[ ]	A statement on whether measurements were taken from distinct samples or whether the same sample was measured repeatedly
[ ]	[x]	The statistical test(s) used AND whether they are one- or two-sided<br>*Only common tests should be described solely by name; describe more complex techniques in the Methods section.*
[ ]	[x]	A description of all covariates tested
[ ]	[x]	A description of any assumptions or corrections, such as tests of normality and adjustment for multiple comparisons
[ ]	[x]	A full description of the statistical parameters including central tendency (e.g. means) or other basic estimates (e.g. regression coefficient) AND variation (e.g. standard deviation) or associated estimates of uncertainty (e.g. confidence intervals)
[ ]	[x]	For null hypothesis testing, the test statistic (e.g. F, t, r) with confidence intervals, effect sizes, degrees of freedom and P value noted<br>*Give P values as exact values whenever suitable.*
[x]	[ ]	For Bayesian analysis, information on the choice of priors and Markov chain Monte Carlo settings
[x]	[ ]	For hierarchical and complex designs, identification of the appropriate level for tests and full reporting of outcomes
[x]	[ ]	Estimates of effect sizes (e.g. Cohen's d, Pearson's r), indicating how they were calculated

Our web collection on statistics for biologists contains articles on many of the points above.

Software and code

Policy information about availability of computer code

Data collection	Graphpad Prism version 9 was used for data collection
Data analysis	Graphpad Prism version 9 was used for data analysis

For manuscripts utilizing custom algorithms or software that are central to the research but not yet described in published literature, software must be made available to editors and reviewers. We strongly encourage code deposition in a community repository (e.g. GitHub). See the Nature Research guidelines for submitting code & software for further information.

Data

Policy information about availability of data

All manuscripts must include a data availability statement. This statement should provide the following information, where applicable:
- Accession codes, unique identifiers, or web links for publicly available datasets
- A list of figures that have associated raw data
- A description of any restrictions on data availability

A data availability statement has been added to the manuscript. Sequencing data of the MA-SARS-CoV-2 genome are deposited to public repository (GISAID accession # EPI_ISL_12243860).

Field-specific reporting

Life sciences study design

All studies must disclose on these points even when the disclosure is negative.

Sample size	sample size was determined based on power calculations performed for previous experiments
Data exclusions	no data was excluded
Replication	Repeated experiments are described individually in the manuscript. Alle repeats were succesfull.
Randomization	Human serum samples were grouped based on their ELISA titers for SARS-CoV-2 Spike antibodies, as described in the text. For animal experiments, animals were allocated to different experimental groups randomly.
Blinding	researchers analysed data blinded, and were blinded for group allocation of animals during data collection. Lung pathology was scored by a pathologist that was blinded to the study design.

Reporting for specific materials, systems and methods

We require information from authors about some types of materials, experimental systems and methods used in many studies. Here, indicate whether each material, system or method listed is relevant to your study. If you are not sure if a list item applies to your research, read the appropriate section before selecting a response.

Materials & experimental systems

n/a	Involved in the study	
☐	☒	Antibodies
☐	☒	Eukaryotic cell lines
☒	☐	Palaeontology and archaeology
☐	☒	Animals and other organisms
☐	☒	Human research participants
☒	☐	Clinical data
☒	☐	Dual use research of concern

Methods

n/a	Involved in the study	
☒	☐	ChIP-seq
☒	☐	Flow cytometry
☒	☐	MRI-based neuroimaging

Antibodies

Antibodies used	Anti-mACE2 (Polyclonal Goat IgG, R&D Systems, Cat# AF3437), Anti-hACE2(Goat polyclonal serum, R&D Systems, Cat# AF944), Anti-Tubulin (Clone 961216, R&D Systems, Cat# MAB9344), Anti-SiglecF (Clone BE50 2440, BD Bioscience, Cat# 552125), anti-Ly6G (Clone 1A8, BD Bioscience, Cat# 562737), anti-EpCAM (Clone G8.8, Biolegend, Cat# 118201), anti-N (1C7C7, in house made), anti-S (2BCE5, in house made), anti-Neutrophil Elastase (polyclonal, abcam, Cat# Ab131260), anti-H3 (polyclonal, abcam, Cat# Ab1791), Ki-67 (Clone 16A8, Biolegend, Cat # 652401), CD11c (Clone N418, Invitrogen, 50-0114-82), CD169 (Cer-4, Invitrogen, Cat #50-5755-82).All antibodies are used at a 1:100 dilution, except for anti-beta-tubulin at 1:1000 dilution. Working dilutions are also reported in the manuscript.
Validation	Primary antibodies are evaluated by flow cytometry or microscopy using A549 (human antibodies) or mouse splenocytes (murine antibodies). For antibodies specific for viral antigens, ELISA is performed using the viral protein as a coating antigen.

Eukaryotic cell lines

Policy information about cell lines

Cell line source(s)	Vero E6 cells were obtained from ATCC
Authentication	Cells were inspected under the microscope and split separately from other cell lines
Mycoplasma contamination	Cell lines and tissue culture aterials are tested weekly for mycoplasma contamination. Only mycoplasma-free cells are used for the experiments described in the manuscript.
Commonly misidentified lines (See ICLAC register)	No commonly misidentified cell line was used in the study.

Animals and other organisms

Policy information about studies involving animals; ARRIVE guidelines recommended for reporting animal research

Laboratory animals	129S1, C57bl6 and Balb/c mice are used for this study. Mice were typically 6-8 weeks of age at the start of the experiment unless differently stated in the text. Mice were housed in a vivarium with controled and monitored ambient temperature and humidity with a 12h day-night light regime.
Wild animals	No wild animals are used for this study.
Field-collected samples	No field collected samples are used for this study.
Ethics oversight	All animal experiments were done adhering to the guidelines from Institutional Animal Care and Use Committee (IACUC) from the Icahn School of Medicine at Mt Sinai.

Note that full information on the approval of the study protocol must also be provided in the manuscript.

Human research participants

Policy information about studies involving human research participants

Population characteristics	A first set of 33 human serum samples was selected from study participants based on their SARS-CoV-2 spike enzyme linked immunosorbent assay (ELISA) antibody titer (Negative [N=4], verus weak [N=8], moderate [N=10] or strong positive [N=11]). In addition, we included sera from six individuals that had received two doses of the Pfizer SARS-CoV-2 vaccine (V1-V6). Demographics and available metadata for each participant is summarized in Supplementary Table 1. A second set of 46 human serum samples was selected in a similar way based on SARS-CoV-2 spike ELISA antibody titers (negative [n=7] versus weak [N=8], moderate [N=13] and strong positive [n=18]. Demographics and metadata for each participant in the second set is summarized in Supplementary Table 2.
Recruitment	Study participants were recruited as part of the PARIS (Protection Associated with Rapid Immunity to SARS-CoV-2) study headed by Dr. Viviana Simon at the Icahn School of Medicine. In this longitudinal study study participants were asked to fill out a survey to report on health status, vaccine status and eventual side effects, and to donate serum/PBMCs at different time points. Patient samples are randomized to avoid selection bias as much as possible.
Ethics oversight	The study protocols for the collection of clinical specimens from individuals with and without SARS-CoV-2 infection by the Personalized Virology Initiative were reviewed and approved by the Mount Sinai Hospital Institutional Review Board (IRB-16-00791; IRB-20-03374). All participants provided informed consent prior to collection of specimens and clinical information. All specimens were coded prior to processing.

Note that full information on the approval of the study protocol must also be provided in the manuscript.
